# Identification of a Novel and Unique Transcription Factor in the Intraerythrocytic Stage of *Plasmodium falciparum*

**DOI:** 10.1371/journal.pone.0074701

**Published:** 2013-09-05

**Authors:** Kanako Komaki-Yasuda, Mitsuru Okuwaki, Kyosuke Nagata, Shin-ichiro Kawazu, Shigeyuki Kano

**Affiliations:** 1 Research Institute, National Center for Global Health and Medicine, Shinjuku-ku, Tokyo, Japan; 2 Graduate School of Comprehensive Human Sciences and Institute of Basic Medical Sciences, University of Tsukuba, Ibaraki, Japan; 3 National Research Center for Protozoan Diseases, Obihiro University of Agriculture and Veterinary Medicine, Obihiro, Japan; Bernhard Nocht Institute for Tropical Medicine, Germany

## Abstract

The mechanisms of stage-specific gene regulation in the malaria parasite *Plasmodium falciparum* are largely unclear, with only a small number of specific regulatory transcription factors (AP2 family) having been identified. In particular, the transcription factors that function in the intraerythrocytic stage remain to be elucidated. Previously, as a model case for stage-specific transcription in the *P. falciparum* intraerythrocytic stage, we analyzed the transcriptional regulation of *pf1-cys-prx*, a trophozoite/schizont-specific gene, and suggested that some nuclear factors bind specifically to the *cis*-element of *pf1-cys-prx* and enhance transcription. In the present study, we purified nuclear factors from parasite nuclear extract by 5 steps of chromatography, and identified a factor termed PREBP. PREBP is not included in the AP2 family, and is a novel protein with four K-homology (KH) domains. The KH domain is known to be found in RNA-binding or single-stranded DNA-binding proteins. PREBP is well conserved in *Plasmodium* species and partially conserved in phylum Apicomplexa. To evaluate the effects of PREBP overexpression, we used a transient overexpression and luciferase assay combined approach. Overexpression of PREBP markedly enhanced luciferase expression under the control of the *pf1-cys-prx cis*-element. These results provide the first evidence of a novel transcription factor that activates the gene expression in the malaria parasite intraerythrocytic stage. These findings enhance our understanding of the evolution of specific transcription machinery in *Plasmodium* and other eukaryotes.

## Introduction

*Plasmodium falciparum* is a significant life-threatening parasitic pathogen that causes falciparum malaria in humans [1]. In spite of years of intensive research, no effective vaccine is presently available, and existing antimalarial drugs are becoming less effective because of the rapid emergence of drug-resistant parasites [2]. To develop new strategies for combating *P. falciparum*, a better understanding of the basic biology of this microorganism is required. In particular, the mechanisms of stage-specific gene regulation remain mostly unclear and only a small number of regulatory transcription factors (TFs) have been predicted in the *Plasmodium* genome [3], [4].

A bioinformatic analysis of the complete *Plasmodium falciparum* genome revealed that the hypothetical Apetala2 (AP2) family with plant AP-2-like DNA-binding domains [5] is the gene family that encodes TF candidates in the *Plasmodium* genome [6], [7]. Twenty-six AP2-related genes were predicted in the *P. falciparum* genome. In a rodent malaria model, it was revealed that some of the AP2 family proteins indeed regulated specific gene expression in the ookinate stage [8], the sporozoite stage [9] and the liver stage [10]. However, the functions of TF in the intraerythrocytic cell cycle remain to be elucidated at the present time.

The intraerythrocytic cell cycle is important for research because it causes the symptoms of malaria [11]. Studies with microarray techniques revealed that at least 60% of the *P. falciparum* genome is transcriptionally active during intraerythrocytic development [12]. The timing of gene expression appeared to be defined quite rigidly and was related to the intraerythrocytic cell cycle of the parasite [12], [13]. As a model of the general transcriptional regulatory mechanisms in *P. falciparum*, we are interested in the mechanism that regulates the transcription of the Pf1-Cys-Prx (PlasmoDB ID, PF3D7_0802200) gene of *P. falciparum* as a model for understanding stage-specific gene expression in the intraerythrocytic stages. Pf1-Cys-Prx is an antioxidant protein and a member of peroxiredoxin family [14]. The expression of *pf1-cys-prx* has been shown to be almost non-existent during the ring stage and markedly elevated during the trophozoite/schizont stage [15]. In a previous study, the authors precisely analyzed the promoter activity of the 5′ region of *pf1-cys-prx*, verified the promoter activities of the 5′ regions of *pf1-cys-prx*, and delineated the 102-bp *cis*-acting enhancer region in this gene [16]. An electrophoresis mobility shift assay (EMSA) and a DNase I footprinting assay both revealed that the double-stranded 102-bp enhancer sequence of *pf1-cys-prx* was the target of trophozoite/schizont stage-specific DNA-binding proteins in the parasite nucleus. We also observed that the levels of histone acetylation in the 5′ region of *pf1-cys-prx* were elevated according to the elevation of transcription. In the *pf1-cys-prx*, a trophozoite/schizont stage-specific recruitment of PfGCN5 histone acetyltransferase to the putative enhancer region was also observed. These results suggest that *P. falciparum* possesses a sophisticated system of transcriptional regulation during the intraerythrocytic stages that is managed by coordinated interactions of unique *cis*-elements, *trans-*acting factors and chromatin modifications.

In the present study, the 102-bp *cis*-enhancer region was defined as Prx Regulatory Element (PRE), and the hypothetical nuclear factor, which binds specifically to the PRE sequence, was defined as PRE Binding Protein (PREBP). PREBP might be a key molecule for the specific transcriptional regulation of *pf1-cys-prx* in the intraerythrocytic stages. We attempted to purify and identify PREBP directly from a huge amount of parasite nuclear extract and finally verified a single protein as authentic PREBP, a novel and unique protein in the *Plasmodium* species, with four K-homology domains that has been found in a number of single-stranded DNA or RNA binding proteins [17], [18]. PREBP is considered to be a novel transcriptional factor, which functions in the intraerythrocytic stages and which is uniquely evolved in the *Plasmodium* species.

## Results

### Purification of *Cis*-Element Binding Protein

**Figure 1:**
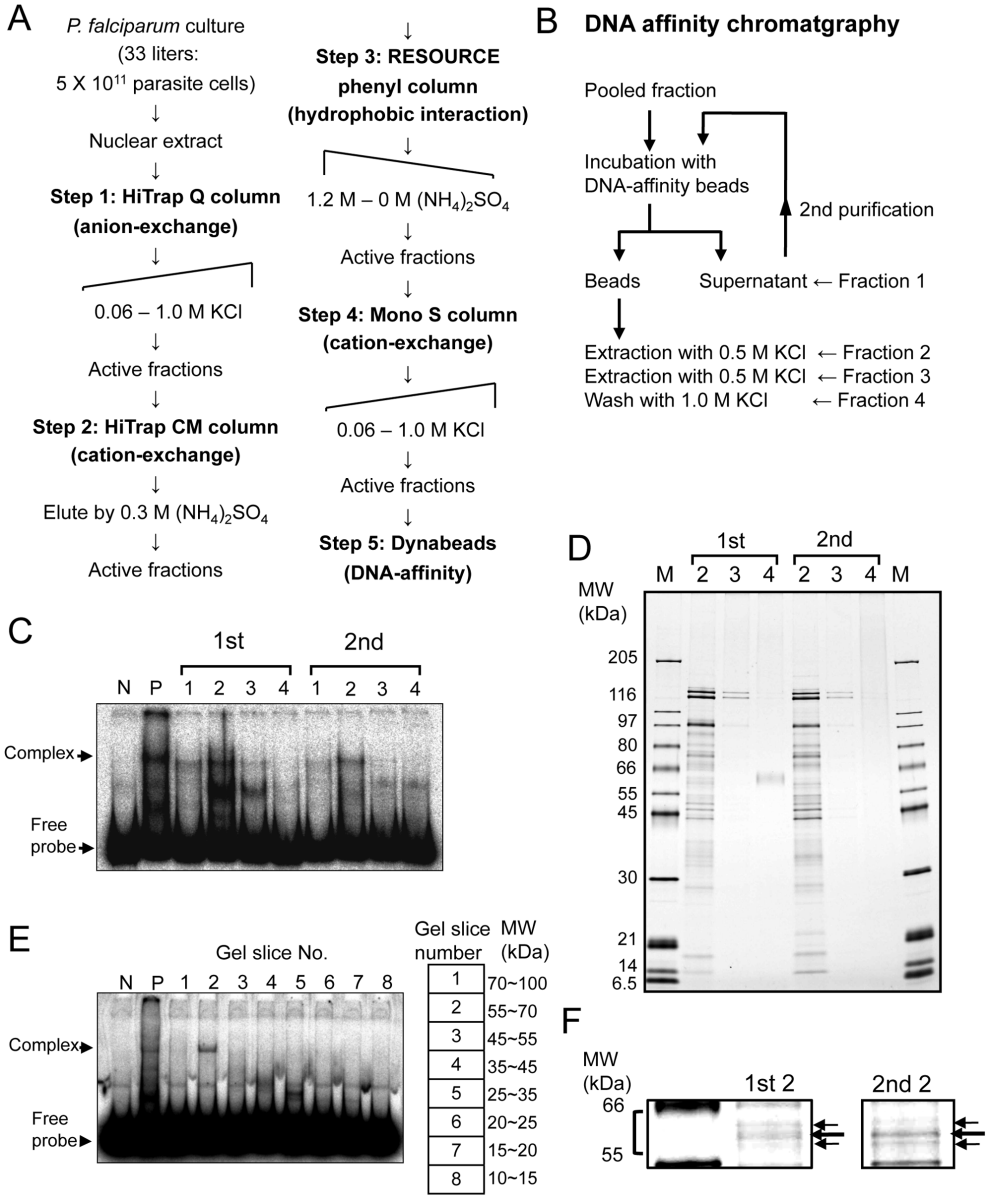
Purification of PREBP. (A) Scheme for purification of PREBP from parasite nuclear extract by 5 chromatography steps. (B) Detailed scheme of the final DNA-affinity chromatography step. (C) EMSA of each elution and washing fraction obtained from DNA-affinity purification. The number of the fraction corresponds to the purification scheme shown in Figure 1B. 0.1 µl of each fraction and the ^32^P-labeled PRE probe were used for the EMSA. Lanes N and P indicate the negative control (probe only) and positive control (assay with the 0.6 µg of parasite nuclear extract). Positions of the free probe and the shifted band are indicated on the left. (D) SDS-PAGE and silver staining of the PRE affinity-purified fractions. 0.5 µl of each PRE-purified fraction was separated on SDS-PAGE and proteins were visualized by silver staining. Molecular mass markers are indicated in kDa on the left. (E) Estimation of the molecular size of PREBP. A partially purified PREBP fraction was applied to an SDS-PAGE. Gel pieces were cut from the gel according to the 8 grades of molecular weight. The range of molecular weight of each gel piece is shown in the right panel. The proteins were eluted from each gel piece and renatured. The PRE-binding activity of the each renatured fraction was checked by EMSA (left panel). (F) Probable candidate bands observed in the final fractions. The 3 candidate bands were indicated with arrows.

In order to identify PREBP, we set up a huge-scale protein purification scheme using 5×10^11^ parasite cells obtained from the culture of 33 liters. PREBP was purified from parasite nuclear extract as summarized in Figure 1A and Table S1. In the final DNA affinity chromatography step, the pooled fraction obtained immediately before Mono S chromatography was incubated with magnetic beads, which tethered the 102-bp PRE sequence (Figure 1B). Each fraction obtained from each DNA affinity chromatography step was checked with EMSA and as a result the first eluents of both the first and second purification step showed specific *cis*-element binding activity (Figure 1C).

The purity of the fractions was assessed by SDS-PAGE followed by silver staining and numerous bands appeared (Figure 1D). Then, to gain insight into the molecular weight of the true binding protein, we performed small-scale protein purification using DNA-coupled magnetic beads. The roughly purified fraction was separated by SDS-PAGE and a series of gel pieces were excised in order of molecular weight (Figure 1E, right panel). Proteins were then eluted from each gel piece and renatured. Each renatured fraction was subjected to EMSA. As a result, DNA-binding activity was detected in the second fraction, corresponding to a molecular weight range of between 55-kDa and 70-kDa (Figure 1E, left panel). In the final fractions of the huge-scale purification, 3 bands were confirmed within the expected molecular weight range (Figure 1F).

### Identification of PREBP by Mass Spectrometry

**Figure 2:**
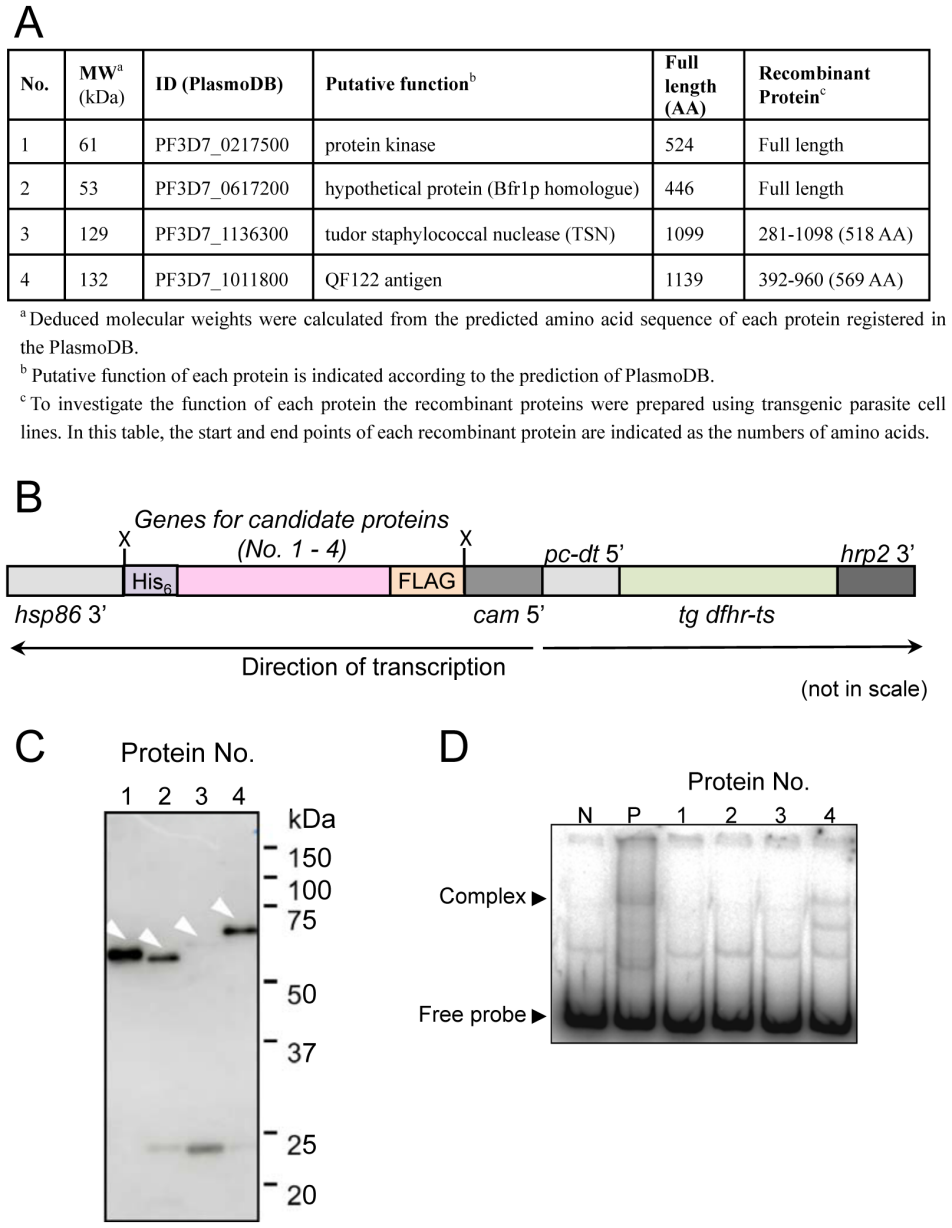
Preparation of the recombinant candidate proteins using gene overexpression system in *P.falciparum*. (A) Detailed descriptions for each candidate protein. (B) Schematic diagram of plasmid constructs used for the gene overexpression in *P. falciparum*. Genes for candidate protein Nos. 1, 2, 3 and 4 were inserted into the expression site of the pHC1 vector. For protein purification, the FLAG-tag and His-tag were added to the N and C termini, respectively. The restriction endonuclease recognition sequences shown is X, *Xho*I. (C) Immunopurification of the recombinant proteins from transgenic parasites. Expressed recombinant proteins were immunopurified with the anti-FLAG antibody and subjected to Western blotting with anti-FLAG antibody. The bands corresponding to the recombinant proteins are indicated with white arrowheads. Sizes are indicated in kDa on the left. (D) EMSA with partially purified recombinant proteins. The same labeled probe was used in Figure 1 in each assay. Lane N is probe only. EMSA was performed with 0.1 µg of each fraction of immunopurified recombinant protein. Lane P is the positive control for the assay with 0.6 µg of nuclear extract derived from the parasite synchronized at the trophozoite/schizont stage. Positions of the free probe and the shifted band are indicated on the left.

**Figure 3:**
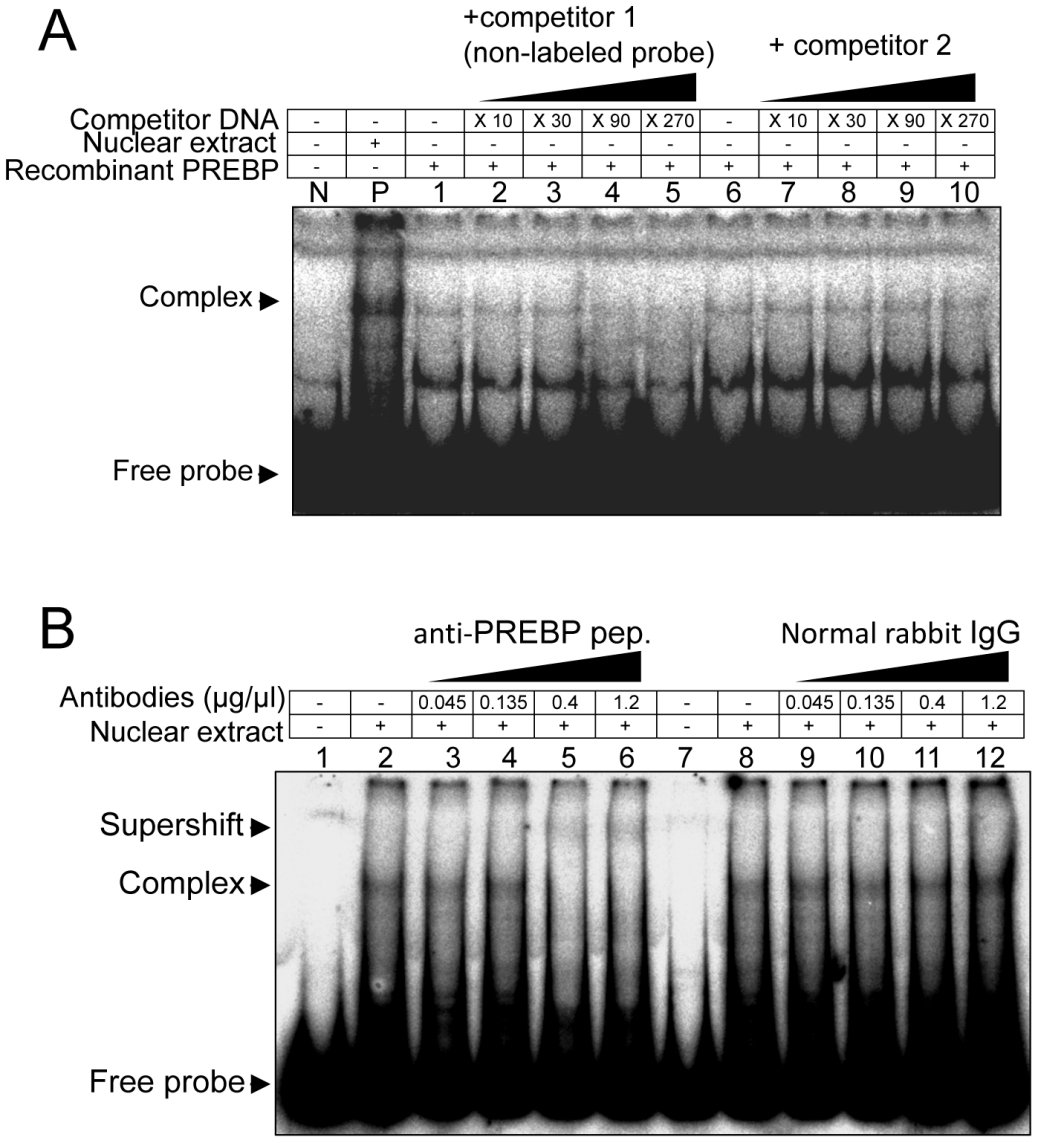
PF3D7_1011800 protein showed specific binding activity to the PRE sequence. (A) Cross-competition assays. EMSA was performed with the same labeled probe used in Figure 1. Lane N is probe only. Lane P is the assay with 0.6 µg of nuclear extract from the parasites at tophozoite/schizont stages. Lanes 1–10 represent EMSA with 0.1 µg of immunopurified PF3D7_1011800 fraction (see Figure 2) in the presence or absence of cross-competitors. Lanes 1–5 represent cross-competition with increasing amounts of non-labeled probe. Lanes 6–10 represent another irrelevant 62-bp sequence [16]. The amount of competitor DNA in each reaction mix was indicated at the top of the panel. (B) Super-shift assays. EMSA was performed with the same ^32^P-labeled probe used in Figure 1. Lane N is probe only. Lane P and Lanes 1–10 represent EMSA with 0.6 µg of nuclear extract from the parasites at tophozoite/schizont stages in the presence or absence of antibodies. The amount of antibody added in each reaction mix is indicated at the top of the panel. Positions of the free probe, shifted band and super-shifted band are indicated on the left.

The final fraction obtained from DNA-affinity chromatography was subjected to SDS-PAGE and visualized by silver staining specialized for mass spectrometry (Figure S1). The 3 bands were excised from the each gel slice, and subjected to mass spectrometry analysis. Database searches with the fragmentation spectra obtained by LC-MS/MS analysis revealed that these bands contained several *P. falciparum* proteins. The 16 proteins that showed the most frequent spectra are indicated in Table S2. To detect true PREBP among these candidate proteins, 16 candidate proteins were first synthesized using a cell-free translation system, and their PRE binding activity was checked by EMSA. However, no recombinant protein showed the specific binding activity (Figure S2, Method S1). Next step, 4 transgenic parasite lines, which expressed protein numbers 1 (PF3D7_0217500), 2 (PF3D7_0617200), 3 (PF3D7_0617200) and 4 (PF3D7_1011800) excessively, were generated (Figure 2A). In the LC/MS/MS analysis, signals corresponding to protein No. 1, 2, 3 and 4 were detected with high frequency and were thus thought to be the 4 most preferable candidates. The expressed recombinant candidate proteins were then roughly purified from the transgenic parasites (Figures 2B and 2C) and their activity was checked by EMSA. Only protein No. 4, which corresponded to PF3D7_1011800, showed specific PRE binding activity and formed a shift-band with similar mobility to that formed with the parasite nuclear extract (Figure 2D). To evaluate the sequence specificity of the interaction, cross-competition assays were performed. The 102-bp sequences of the PRE or other AT-rich DNA fragments derived from the 5′ region of the *pf1-cys-prx* were added to the EMSA reaction mixtures as competitors. A 90-fold molar excess of unlabeled probe prevented the formation of DNA-protein complex (Figure 3A, lane 4). It is noteworthy that the 270-fold molar excess of AT-rich fragments obtained from the 5′ regions of *pf1-cys-prx* showed less inhibition of DNA-protein complex formation. Thus, this fragment could not completely prevent a band shift. These results indicated that the interaction between the PRE and the recombinant PF3D_1011800 was sequence-specific. Furthermore, the peptide-antibody against the PF3D_1011800 candidate protein was raised (anti-PREBP pep.) and added into the reaction mixtures of EMSA using the parasite nuclear extract and the 102-bp PRE sequence as a labeled probe, which resulted in a band-shift corresponding to the complex of the probe and authentic PREBP in the nuclear extract. The band-shift disappeared and a super-shift band appeared when the antibody was added into the reaction mix, indicating that the antibody against the PF3D7_1011800 protein bound specifically to the PREBP-*cis*-element complex (Figure 3B). Thus, PF3D7_1011800 was verified as an authentic PREBP.

### Molecular Features of the Identified PREBP, PF3D7_1011800

**Figure 4:**
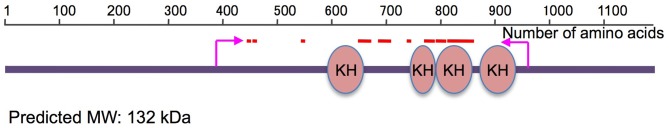
The predicted structure of the verified PREBP, PF3D7_1011800. Domains within the PF3D7_1011800 protein predicted from its amino acids sequence. The peptides detected in mass spectrometry are indicated with red bars. The predicted K-homology (KH) domains are indicated with circles. The start and end points of the partial ∼60 kDa recombinant protein, which is expressed in the transgenic parasite, are indicated as arrows.

The amino acid sequence and characteristic features of the PF3D7_1011800 are summarized in Figure S3 and Figure 4. In PlasmoDB, PF3D7_1011800 is registered as “QF122 antigen” [19]. The deduced molecular mass of PREBP was 132 kDa. The size of PREBP estimated by SDS-PAGE was ∼60 kDa (Figure 1F). The peptides detected by LC-MS/MS covered the central 60 kDa region of PF3D7_1011800. No peptides corresponding to the N or C terminal regions were detected. Thus, the band of ∼60 kDa obtained from the purification steps (Figure 1F) was deduced to be a shortened form due to the processing of protein in the parasite cells, or artificially cut form that can be produced during the purification steps. The characteristic feature of PREBP is that it contains four putative K-homology (KH) domains (Figure 4). The orthologs of PREBP are highly conserved in *Plasmodium* species and those with lower identity have been found in many other Apicomplexan parasites, *Cryptosporidium*, *Theileria* and *Bavesia* (Table 1). No ortholog was found in the *Toxoplasma* genome. The PREBP mRNA expression profile during intraerythrocytic development was analyzed by real-time RT-PCR using cDNA obtained from tightly synchronized parasites. PREBP mRNA expression was almost constant during the intraerythrocytic development, although only slight changes of expression were observed (Figure S4, Method S2).

**Table 1:** The orthologs of PREBP in other *Plasmodium* and Apicomplexan spp.

Organ	Gene ID^a^	Protein length^b^	Similarity (%)^c^	Identity (%)
*P. yoelii*	PY03523	979	91.7	60.7
*P. berghei*	PBANKA_121020	970	90.7	61.0
*P. chabaudi*	PCHAS_121090	975	92.3	62.3
*P. vivax*	PVX_094810	985	90.5	54.6
*P. knowlesi*	PKH_081130	993	88.9	54.7
*P. cynomologi*	PCYB_082160	964	88.5	52.8
*Cryptosporidium parvum*	EAK90228.1	818	68.8	16.6
*Theileria perve*	EAN34110.1	864	65.8	16.2
*Bavesia bovis*	EDO07257.1	850	70.6	17.1

a Gene ID is the PlasmoDB ID for *Plasmodium* genes and Genbank ID for other Apicomplexan genes.

b Protein length is number of amino acids.

c Similarity and identity are calculated by comparison with the PREBP of *P. falciparum.*

### Endogenous PREBP in the Trophozoite/Schizont Nucleus was Expressed as Proteins of Approximately 132 kDa

**Figure 5:**
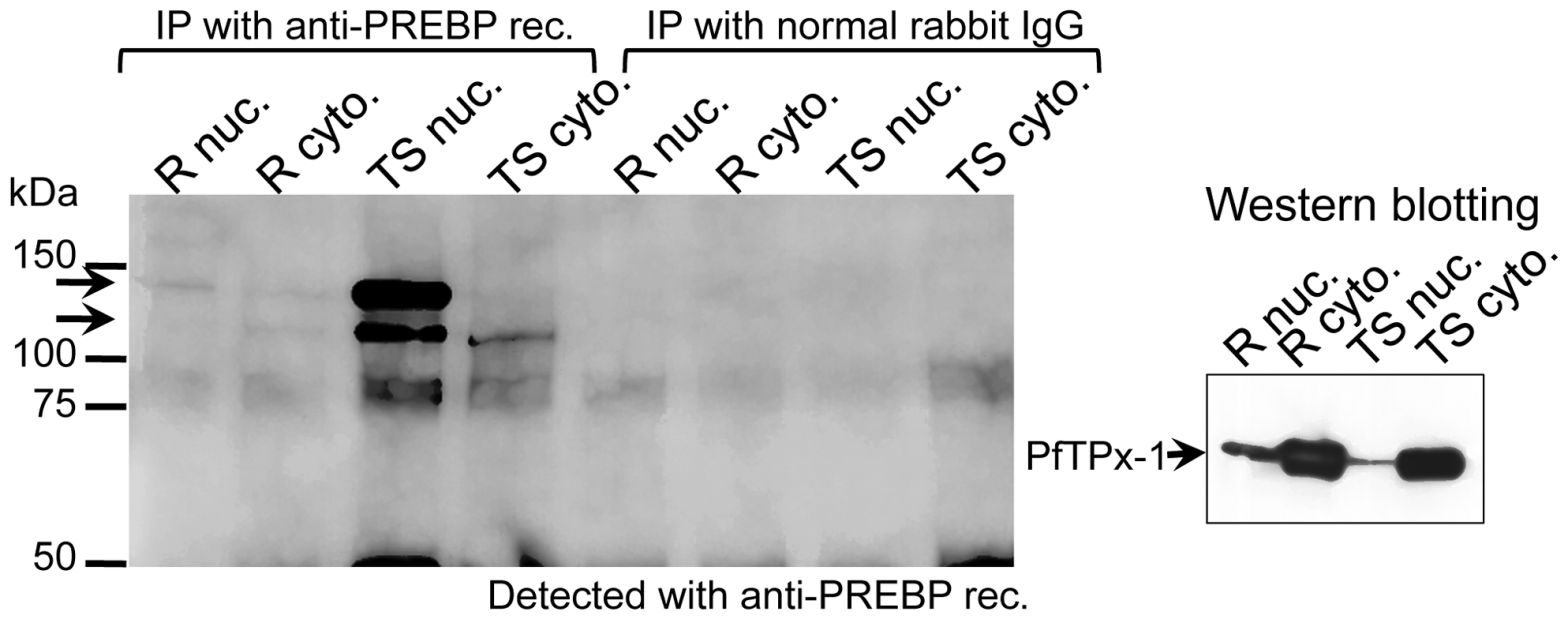
Endogenous expression and cellular localization of PREBP protein. Left panel: Nuclear extract from trophozoite/schizont parasites was subjected to immunoprecipitation (IP) with anti-PREBP rec. or Normal Rabbit IgG. The IP products were separated in SDS-PAGE and blotted with the anti-PREBP rec. Arrows are shown to denote the bands considered to be endogenous PREBP. Right panel: Western blot with the PfTPx-1 antibody. To test the fractionation efficiency of each sample used for the IP-Western, 2 µg of each fraction was separated in 5–20% SDS-PAGE and blotted with the specific antiserum for PfTPx-1, a constitutive expressed cytoplasmic protein [15]. R nuc., R cyto., TS nuc., and TS cyto. indicate nuclear extract from ring stage parasites, cytoplasmic fraction from ring stage parasites, nuclear extract from trophozoite/schizont stage parasites and cytoplasmic fraction from trophozoite/schizont stage parasites, respectively.

To detect the stage-specific expression and cellular localization of the endogenous PREBP protein, IP-Western blotting experiments with nuclear extract and cytoplasmic fraction prepared from both ring and trophozoite/schizont parasites were performed (Figure 5). For this purpose, antibody was raised against recombinant partial PREBP protein, which corresponded to amino acid numbers 699–849 (anti-PREBP rec.). As a result, 2 bands of ∼130 kDa were detected in the anti-PREBP antibody-precipitant from the nuclear extract of trophozoites/schizont parasites. From the nuclear extract and cytoplasmic fraction of ring stage parasites, only faint bands were detected in the anti-PREBP antibody-precipitant. From the cytoplasmic fraction of trophozoite/shizont parasites, only a sub-band of higher mobility was detected. Thus, most endogenous PREBP protein was localized at the nucleus of trophozoite/schizont parasites. No other band was detected at 60∼70 kDa in any sample. Thus, it is suggested that endogenous PREBP exits in the parasite nucleus as ∼132 kDa proteins, which correspond to the full length deduced from the sequence of ORF registered in PlasmoDB. The ∼60 kDa band obtained from purification steps (Figure 1) was deduced to be an artificially cut and shortened form through the purification steps. The expression level of *pf1-cys-prx* in the trophozoite/schizont stages of the PREBP central ∼60 kDa overexpressor was quantified by real-time RT-PCR and compared with that of the parental line. No significant difference was observed between the transgenic and parental parasites (Figure S5, Method S2). Furthermore, to investigate the effect of overexpression of full-length PREBP, a plasmid construct for the excessive expression of full-length PREBP was transfected into the parasite. To obtain stable overexpressor, transgenic parasites with episomal plasmids were selected by pyrimethamine pressure. However, in spite of repetitive trials, no living transgenic parasite was obtained after drug selection (data not shown).

### Overexpression of PREBP Enhanced the Expression of Reporter Gene Depending on the PRE Sequence in a Transient Luciferase Assay

**Figure 6:**
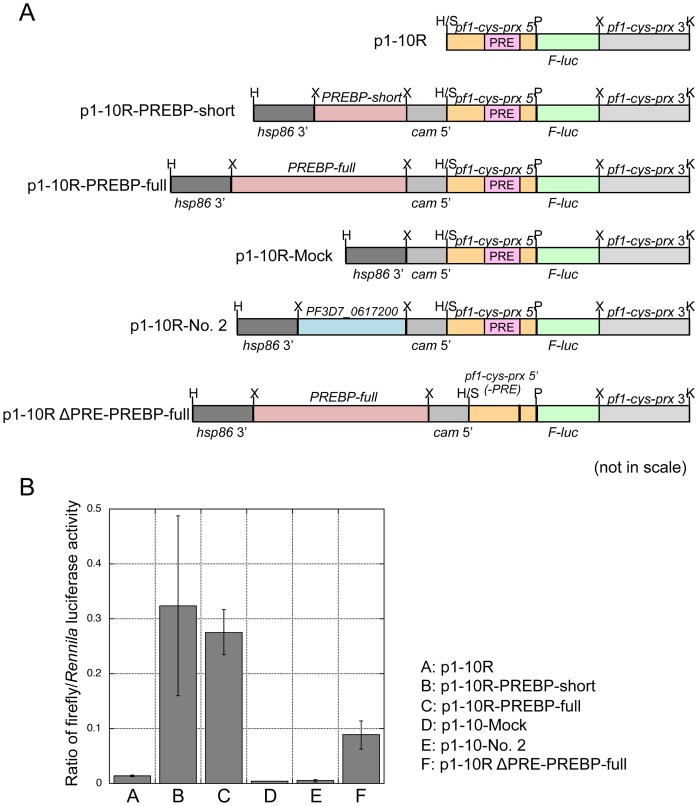
PREBP is critical for the expression of the reporter gene under the *pf1-cys-prx* promoter. (A) Schematic diagram of plasmid constructs used for the transient luciferase assay in *P. falciparum*. The restriction endonuclease recognition sequences shown are E, *EcoR*I; H, *Hind*III; K, *Kpn*I; P, *Pst*I; S, *Sbf*I and X, *Xho*I. (B) The dual luciferase assay. The plasmids shown in Figure 6A were transfected into the parasite synchronized in ring stage. For the dual luciferase assay the pHC1-Rluc plasmid, which expresses Rennila luciferase, was co-transfected in each assay. After 20–24 h, the luciferase activities of the transfected parasites were measured and the ratio of firefly/Rennila luciferase activity was calculated. The data are indicated as means of three independent assays with standard deviation.

To evaluate the effects of overexpression of full-length PREBP, we combined transient overexpression and a luciferase assay. The plasmid constructs used for the assays are indicated in Figure 6A. Plasmid p1-10R was the basal construct, which contains a firefly luciferase gene expression cassette controlled under the 0.8-kbp 5′ region of the *pf1-cys-prx* gene. In p1–10R-PREBP-full and p1-10R-PREBP-short, the expression cassette for full-length PREBP and partial ∼60 kDa PREBP, respectively, were also inserted into the basal p1–10R under the control of 5′ of calmodulin. Luciferase expression in the p1–10R-PREBP-full transfected cell would be expected to be enhanced if the overexpressed full-length PREBP affected the 5′ promoter sequence of *pf1-cys-prx* gene on the same plasmid. Although the transfection efficiency of the electroporation system for *P. falciparum* is very low [20], enhanced expression of luciferase could be detected by the luciferase assay system because of its high sensitivity. As negative controls, we prepared p1–10R-Mock, which contained an empty overexpression cassette and p1-10R-No.2, which contained a candidate protein No. 2 (PF3D7^_^0617200: hypothetical Bfr1p homologue) overexpression cassette. These plasmid constructs were introduced into parasites synchronized in the ring stage by eletroporation. After 20–24 hours, luciferase activity was determined at the trophozoite/schizont stages, before the completion of the cell cycle after transformation. Parasites transfected with p1–10R-PREBP-short or p1–10R-PREBP-full showed clear and drastic elevation of the reporter gene in comparison to parasites transfected with the basal plasmid, p1–10R (*P*<0.01). In contrast, no detectable elevation of reporter activity was observed in parasites transfected with p1–10R-Mock or p1–10R-No.2 (Figure 6B). These results suggest that in the parasites transfected with p1–10R-PREBP-full or p1–10R-PERBP-short, the overexpressed PREBP proteins affected the 5′ sequence of *pf1-cys-prx* and enhanced the expression of the reporter gene (Figure S6: model scheme).

Furthermore, to investigate the dependency of the 102-bp PRE sequence on the enhancing activity of PREBP, a mutant plasmid of p1–10R-PREBP-full, which lacked the 102-bp PRE sequence of the 5′ region of *pf1-cys-prx* gene, was constructed (p1–10R-Δcis-PREBP-full) and transfected into the parasite. As a result, reporter activity of the parasites transfected with the mutant plasmid, p1–10R-Δcis-PREBP-full, decreased to less than 30% of the reporter activity of the transfectant of the original plasmid, 1–10R-PREBP-full (*P*<0.01, Figure 6B).

## Discussion

Here, we report for the first time the purification and identification of a specific *cis*-element binding protein from the malaria parasite *P. falciparum*. The identified PREBP (PF3D7_1011800) binds the 102-bp PRE sequences with high affinity. In addition, we show that the overexpression of PREBP enhances the expression of the reporter gene under the control of the 5′ sequence of *pf1-cys-prx* sequence using the transient luciferase assay system and that this enhancing activity is dependent on the 102-bp PRE sequence. This result strongly suggests that the PREBP is a novel transcription factor in *Plasmodium*.

Despite exhaustive research on transcriptomes and proteomes based on the genome database of *Plasmodium* [12], [13], [21], [22], little has been known about the regulation mechanisms of gene expression in the parasite [3], [4]. The paucity of the knowledge of the basic biology might reflect the phylogenic distance of the parasites from other eukaryotes. The phylum Apicomplexa, which is composed of a number of protozoan parasites including *Plasmodium*, belongs to the Alveolate phylogenetic group, the members of which are vastly distant from members of both the Opisthokonta (including most of model eukaryotes from yeasts to humans) and Planta branches [23].

The identified PREBP was registered as “QF122 antigen” in the PlasmoDB database because the partial sequence of this protein was determined in a previous report [19]. The screening of the parasite cDNA expression library with a monoclonal antibody bound to the merozoite surface and Western blotting showed that this monoclonal antibody recognized the 51 kDa protein in the parasite cell lysate. However, no follow-up was reported for this “antigen” The detected PREBP was not a member of AP2 family, which is the only known specific transcription factor in *Plasmodium* [6], [7], [8], [9], [10]. Rather, it was a novel protein with four K-homology (KH) domains. The KH domain was first identified in human heterogeneous nuclear ribonucleoprotein (hnRNP) K [24]. The KH motif consists of approximately 70 amino acids, and is found in a diverse variety of proteins in archaea, bacteria and eukaryota [17], [24]. It is known that the KH domain is contained in a number of RNA or single-strand DNA binding proteins that perform wide range of cellular functions [17], [18]. Previously, it had been reported that a human protein with a KH domain could function as a transcription factor to activate the expression of the human *c-myc* gene [25]. This transcription factor (FBP) activates gene expression by binding to the single-stranded *cis*-element (FUSE: Far UpStream Element) [25], [26]. In our previous study, we showed that the authentic PREBP in the nuclear extract could interact with the double-stranded *cis*-element sequence by DNaseI footprinting assay [16]. Thus, this study is the first report that shows that a protein with a KH domain functions as a transcription factor, which binds to specific double-stranded *cis*-element sequence. In future studies, it will be informative to elucidate whether PREBP binds to single-stranded-DNA and RNA in addition to the double-stranded PRE sequence. With the exception of *Plasmodium* species and some Apicomplexan species, no PREBP orthologs have been found in any other organisms. These findings indicate that this factor evolved uniquely in the *Plasmodium* species.

The mRNA expression of PREBP was not trophozoite/schizont stage-specific during the intraerythrocytic stages. However, the expression of PREBP protein was increased at the trophozoite/scizont stages, suggesting that the expression of PREBP protein is regulated by post-transcriptional mechanisms. In addition, the immunoprecipitation-Western blotting analyses represented two bands of approximately 130 kDa as the endogenous PREBP proteins in the nuclear extract from trophozoite/schizont parasites. In the cytoplasmic fraction from trophozoite/schizont parasites, only the band with higher mobility was detected. These two bands with slight differences in mobility might reflect the existence of a modified form of the PREBP protein, which may permit the lower mobility protein to localize specifically at the parasite nucleus.

The stable overexpression of the partial ∼60 kDa sequence of PREBP did not affect the expression of *pf1-cys-prx* gene; however, transgenic parasites which excessively expressed full-length PREBP could not be established. Since neither the overexpression nor the disruption of *pf1-cys-prx* gene affected parasite growth [27], [28], this observation suggests that the overexpression of full-length PREBP might disrupt the balance of not only *pf1-cys-prx*, but also the expression of many other genes in the parasite cell and have a lethal effect on the parasite physiology. We tested the effects of overexpression of full-length PREBP using the transient luciferase assay system and the results clearly showed that overexpression of full-length PREBP enhances the expression of the reporter gene under the control of the 5′ sequence of *pf1-cys-prx*. Unexpectedly, partial ∼60 kDa PREBP also enhanced the expression of the reporter gene under the promoter of *pf1-cys-prx*, despite the lack of detection of any significant affection in the transgenic parasites that stably overexpressed partial ∼60 kDa PREBP. In the transient reporter assay system, the introduced plasmid DNA is considered to not be a formed chromatin structure. Thus, our results might suggest that full-length PREBP controls the expression of the endogenous gene in the chromatin structure. Partial ∼60 kDa PREBP might bind the naked DNA sequence of PRE, but may not be able to access the *cis*-element of the endogenous gene because of its chromatin structure. The N and C terminal regions of PREBP protein might associate with other chromatin remodeling factors such as histone acetyl transferases and reconstitute the *cis-*element as an accessible form. Our previous study showed that the level of histone acetylation of the promoter region of *pf1-cys-prx* gene was elevated at the trophozoite/schizont stages according to the activation of gene expression, and that the PfGCN5 protein, the histone acetyltransferase of *P. falciparum* [29], was recruited into the PRE sequence [16]. It has been suggested that the mechanisms of histone modification and epigenetic regulation for transcription are as well conserved in the parasite as they are in other eukaryotes [30], and that the degree of histone acetylation reflects the activity of transcription [16], [31]. The PREBP protein might interact with the PfGCN5 and regulate the chromatin structure for the effective activation of gene expression.

In the transient reporter assay, the transfectant with the plasmid p1–10R-Δcis-PREBP-full showed a significant decrease in firefly luciferase activity, indicating that the enhancer activity of PREBP depends on the PRE sequence. However, this level of decrease was not as low as that in the transfectant of p1–10R-Mock and p1–10R-No.2. This some elevation of expression might be the effect of non-specific binding of PREBP to the promoter sequence in the plasmid, which does not form a chromatin structure. Our previous report described the measurement of the promoter activity of the *pf1-cys-prx* 5′ sequence with and without the PRE element [16]. The plasmids used in the previous study did not express PREBP. The promoter without PRE showed about 70% of promoter activity of that with the PRE element. Thus, it is suggested that the PRE element was more effective with an excess of PREBP.

It is also possible that PREBP regulates the numbers of genes other than *pf1-cys-prx*. In mammals, the expression of 1-Cys-Prx is induced by oxidative stresses through the function of the antioxidant response element in the promoter [32]. Oxidative stress, however, could not enhance the expression of the *pf1-cys-prx* gene (Komaki-Yasuda K, unpublished data) in the intraerythrocytic stage. Thus, its expression is rigidly regulated in accordance with the progression of the parasite cell cycle in the intraerythrocytic stages [12], [13], [15]. The expression of other genes regulated by PREBP might be elevated at the same time-point in the trophozoite/schizont stages. In future studies, it would be interesting to use ChIP-seq methods to discover the genes that are regulated by PREBP binding [33]. The structural analyses would elucidate the means by which PREBP recognizes and specifically binds to the sequence of *cis-*element in the AT-rich non-coding DNA sequence in *P. falciparum*. It would also be of great interest to detect the co-factors that function with PREBP in the process of transcriptional activation.

It is known that an enormous variety of specific transcription factors is present across different eukaryotic lineages [4], [34]. Thus, only a small number of specific transcription factors have been identified in *Plasmodium* spp., and across the range of protist species [4]. The discovery of PREBP showed the possibility that unknown specific transcription factors with unique DNA binding domain had evolved in the protists. As a model case of the general transcriptional regulatory mechanisms in *P. falciparum*, further analysis of the transcriptional activation of *pf1-cys-prx* will yield profound insights into the gene activation mechanisms present during the intraerythrocytic growth of the parasite. Further detailed characterization of novel components of the transcriptional machinery in malaria parasites may help lead to the black box of evolution of specific transcription machinery in the protists, and may pave the way for the identification of unique drug targets for alternative malaria chemotherapies.

## Materials and Methods

### Parasite Culture

The FCR-3 strain of *P. falciparum* was cultured in human O erythrocytes and RPMI medium (Life Technologies, Carlsbad, CA) supplemented with 10% human serum using the modified method of Trager and Jensen [35]. For the preparation of nuclear extracts, parasites were cultured in RPMI medium supplemented with 0.5% AlbumaxI (Life Technologies). Growth synchronization was achieved with 5% D-sorbitol [36].

### Parasite Nuclear Extracts and Cytoplasmic Fractions

Parasite nuclear extracts were prepared with modifications according to the previous study [37]. In brief, parasites were released from red blood cells by treatment with PBS containing 0.05% saponin and washed twice in PBS. The parasite pellet was resuspended in ice-cold lysis buffer (20 mM Hepes, pH 7.8, 10 mM KCl, 1 mM EDTA, 1 mM DTT, 1 mM PMSF and 0.65% Nonidet P-40) and incubated for 5 min on ice. Nuclei were pelleted at 2,500×*g* for 5 min, and the supernatants were saved as cytoplasmic fractions. The nuclear pellet was washed twice with lysis buffer before resuspension in one pellet volume of extraction buffer (20 mM Hepes, pH 7.8, 800 mM KCl, 1 mM EDTA, 1 mM DTT and 1 mM PMSF) containing Complete Protease Inhibitor Cocktail (Roche Applied Science). After vigorous shaking at 4°C for 30 min, the extract was cleared by centrifugation at 13,000×*g* for 30 min. The supernatant (nuclear proteins) was diluted with an equal volume of dilution buffer (20 mM Hepes, pH 7.8, 1 mM EDTA, 1 mM DTT and 30% glycerol), then immediately frozen in liquid nitrogen and stored at−80°C.

### Electrophoretic Mobility Shift Assay (EMSA)

To generate the labeled probe for EMSA, the 102-bp putative enhancer sequence (PRE: region between 490–591 bp upstream of the ATG translational start site of *pf1-cys-prx*) in the 5′ region of *pf1-cys-prx* was labeled with [γ-^32^P]ATP with T4 polynucleotide kinase as described previously [16]. For EMSA reactions, each protein sample was incubated with 10 fmol of labeled probe in EMSA buffer (20 mM Hepes, pH 7.8, 60 mM KCl, 0.5 mM EDTA, 2 mM DTT, 0.1% Triton X-100 and 10% glycerol) in a 20 µl reaction volume for 20 min at 25°C. To decrease non-specific binding, 0–0.025 µg/ µl of poly-dI-dC was added to the reaction mix. To stabilize the DNA-protein complex, 0–0.0125 µg/µl of poly-L-lysine was also added to the reaction mix. Binding reactions were analyzed by 6% polyacrylamide gel electrophoresis in 0.25 × TBE buffer at 4°C. For the super-shift assay, 0.9–27.0 µg of antibodies was added into the reaction mix of EMSA and incubated overnight at 4°C before electrophoresis.

Gels were dried, and the result was visualized using a BAS-2000 system (Fuji Film, Tokyo, Japan).

### Purification of the Specific DNA-Binding Protein

Throughout all of the purification steps, the activities of PREBP were checked by EMSA with the labeled PRE sequence. The frozen stock of nuclear extract (110 ml, obtained from 5 × 10^11^ parasite cells synchronized at trophozoite/schizont stage) was thawed and dialyzed against buffer 1 (60 mM KCl, 20 mM Hepes (pH 7.8), 0.5 mM EDTA, 2 mM DTT, 0.1% Triton X-100, 10% glycerol, 1 mM PMSF). The dialyzed nuclear extract was loaded onto HiPrep Q column (GE Healthcare, Buckinghamshire, UK) with an HPLC system (AKTA Prime, GE healthcare). The column was washed with the same buffer and the bound materials were eluted with 200 ml of an increasing (60–1000 mM) KCl gradient. Activity was eluted at ∼0.3 M KCl. Active fractions were pooled, diluted with KCl-free buffer 1 until they reached 60 mM KCl concentration, and loaded onto the HiTrap CM column (GE Healthcare) using the AKTA prime system. After the column was washed with buffer 1, the bound protein was eluted with stepwise increasing Na_2_SO_4_ (50 ml of buffer 2 (0.3 M Na_2_SO_4_, 20 mM Hepes (pH 7.8), 0.5 mM EDTA, 2 mM DTT, 0.1% Triton X-100, 10% glycerol, 1 mM PMSF), 50 ml of buffer 2 with 0.6 M Na_2_SO_4_ and 50 mM of buffer 2 with 1.2 M Na_2_SO_4_). Active fractions (with 0.3 M Na_2_SO_4_) were pooled and buffer 2 was added with 3 M Na_2_SO_4_ to reach 1.2 M Na_2_SO_4_ concentration and then loaded onto a RESOURCE PHE column (GE Healthcare) using the AKTA Prime system. The column was washed with buffer 2 containing 1.2 M Na_2_SO_4_ and the bound protein was eluted with 20 ml of a decreasing (from 1.2 to 0.1 M) Na_2_SO_4_ gradient and 10 ml of buffer 2 containing 0.1 M Na_2_SO_4_. The active fractions (with ∼0.1 M Na_2_SO_4_) were pooled and dialyzed against buffer 1, then loaded onto a Mono S column (GE Healthcare) using the SMART system (GE Healthcare). The bound proteins were eluted with 3 ml of a linear gradient from 60 to 1000 M KCl. Active fractions (with ∼0.3 M KCl) were pooled, diluted to 60 mM KCl concentration and applied to the DNA affinity purification.

As a matrix for affinity purification, the biotinylated PRE DNA sequence was tethered to magnetic streptavidin-coated Dynabeads M280 (Life Technologies) according to the supplier’s instructions. The dialyzed fractions were supplied with 25 µg/ml of poly(dI-dC) and 2.5 µg/ml of poly-L-lysine, then incubated with 0.5 mg of Dynabeads (100 pmol oligo/mg beads) on a rotating wheel for 3 h. Dynabeads were collected using a magnetic stand and washed twice with 1 ml of wash buffer 1. Bound proteins were eluted from the beads with 10 µl of elution buffer 1 (20 mM Hepes, pH 7.8, 500 mM KCl, 1 mM EDTA, 1 mM DTT, 1 mM PMSF, 0.01% Triton X-100, 5% glycerol) twice and 10 µl of elution buffer 2 (elution buffer 1 containing 1.0 M KCl). The flow through fraction was pooled and applied onto the DNA-tethered Dynabeads, and the same washing and elution process was repeated twice.

### Estimation of the Molecular Size of PREBP

Two hundred µl of parasite nuclear extract was dialyzed against buffer 1 (60 mM KCl, 20 mM Hepes (pH 7.8), 0.5 mM EDTA, 2 mM DTT, 0.1% Triton X-100, 10% glycerol, 1 mM PMSF), added to 25 µg/ml of poly(dI-dC) and 2.5 µg/ml of poly-L-lysine, and treated with 0.2 mg of the DNA-affinity magnetic beads tethered to the 102-bp PRE sequence. The bound protein was eluted with 8 µl of the buffer 1 with 0.5 M of KCl, and applied to 5–20% SDS-PAGE. The gel pieces were cut from the gel according to the 8 grades of molecular weights, ranging 10–100 kDa. The proteins were eluted from each gel piece by treatment with 200 µl of elution buffer (50 mM Tris pH7.9, 150 mM NaCl, 0.1 mM EDTA, 0.1% SDS, 0.05 mg/ml BSA), precipitated with 80% of acetone, and the precipitated proteins were dissolved in 100 µl of the buffer (50 mM Hepe7.9, 10 mM DTT, 6 M Guanidine HCl). The proteins were then renatured by removing the guanidine HCl by dialysis against buffer 1. To concentrate the DNA binding protein, each renaturated fraction was supplied with 25 µg/ml of poly(dI-dC) and 2.5 µg/ml of poly-L-lysine, then again treated with 0.02 mg of the DNA-affinity magnetic beads tethered to the 102-bp PRE sequence. The bound protein was eluted with 2 µl of the buffer 1 with 0.5 M of KCl. The PRE-binding activity of each fraction was checked by EMSA.

### Mass Spectrometry and Data Analysis

Seven µl of the final fraction obtained from the purification steps was subjected to 5–20% SDS-PAGE and stained with silver stain system specialized for mass spectrometry (SilverQuest, Life Technologies). The 3 candidate bands were excised as gel slices, digested with trypsin, and analyzed by nanoliquid chromatography and mass spectrometry on an LTQ-Orbitrap XL tandem mass spectrometer (Thermo Fisher Scientific, San Jose, CA). Data were searched against the Mascot concatenated forward-and-reversed v3.46 International Protein Index database (Matrix Science, London, UK) and collated into nonredundant lists using Scaffold software ((Proteome Software, Portland, OR).

### Antibodies

Rabbit polyclonal antiserum was raised against a cocktail of two synthetic peptides (CGMKDDIENLKELIE and C+ELNISGNKNDIDKA, corresponding to the amino acid numbers 568–582 and 770–783 (“C+” indicates that a cysteine residue was added at the N terminus for coupling to carrier keyhole limpet hemocyanin), respectively, of the predicted PF3D_1011800 sequence) and affinity-purified as described previously [16]. The purified antibody was defined as anti-PREBP pep. In addition, Rabbit polyclonal antiserum was raised against the partial recombinant PREBP protein. For the antigen, recombinant partial PREBP 17 kDa protein (corresponding to amino acid numbers 699–849) was expressed as a glutathione S-transferase (GST) fusion protein with pGEX-6P-1 vector system (GE Healthcare) and *Escherichia coli* BL21 strain. The GST-tag on the fusion protein was removed by PreScission protease treatment and GST-glutathione affinity chromatography (GE Healthcare). The recombinant protein was further purified with gel filtration chromatography with a HiPrep Sephacryl S-100 column (GE Healthcare) and used for rabbit immunization. The antibodies were affinity-purified with the HiTrap NHS-activated HP column (GE Helthcare) coupled with the recombinant PREBP protein (the antigen) according to the manufacturer’s instructions. The purified antibody was defined as anti-PREBP rec. The antibody against FLAG (Sigma-Aldrich, St. Louis, MO) was commercially supplied.

### Construction of Plasmids

For construction of plasmid vectors for the overexpression of the candidate proteins, each gene was first amplified by PCR with the parasite cDNA as a template. The sequence of each primer used for the PCR is indicated in Table S3. FLAG-1F and His-1R were used for protein No. 1; FLAG-2F and His-2R were used for protein No. 2; FLAG-3F and His-3R were used for protein No. 3; and FLAG-4F and His-4R were used for protein No. 4. These PCR products contain the sequences for the FLAG-tag and His-tag at the 5′ and 3′ ends, respectively. For insertion of these fragments into the pHC1 expression vector (MRA-4, Malaria Research and Reference Reagent Resource [MR4] Center [38]), the *Xho*I sites were added at both the 5′ and 3′ ends by reamplification of these fragments by PCR using the primers FLAG-XhoI and His-XhoI. These fragments were then inserted into the *Xho*I site of the pHC1 and the constructed plasmids were termed pHC1-No.1, pHC1-No.2, pHC1-No.3 and pHC1-No.4. The direction of the inserted fragments was checked by sequencing analysis.

For the overexpression of full-length PREBP, the complete ORF of PREBP gene was amplified using the primers, and PREBP-F and PREBP-R with the parasite cDNA as a template. The amplified fragment contained FLAG-tag at the 5′ end, HA tag at the 3′ end and the *Xho*I sites at both the 5′ and 3′ ends. The fragments were inserted into the *Xho*I site of pHC1. Furthermore, for the following operation of the plasmid, the *Hind*III site in the ORF was disrupted by a Site-Directed Mutagenesis System (Promega), avoiding the non-synonymous substitution of amino acid, with the primers ΔHindIII-F and ΔHindIII-R. This plasmid was termed pHC1-PREBP-full. The direction of the inserted fragments was checked by sequencing analysis.

For the transient luciferase assays, a basal plasmid, p1–10R was first constructed. The luciferase expression cassette was amplified by PCR with the primers 1-cys5′-F and 1-cys3′-R. The p1cys-Fluc plasmid [16] was used as a template. This fragment contains 0.8-kbp of the 5′ region of *pf1-cys-prx*, 1.7-kb of firefly luciferase gene and 0.9-kb of 3′ region of *pf1-cys-prx*. This fragment was digested by *Sbf*I and *Kpn*I and subcloned into the same restriction site of PUC19.

To obtain the p1–10R-PREBP-short, p1–10R-PREBP-full, p1–10R-Mock and p1–10R-No.2, the expression cassettes were excised from pHC1-No.4 (for p1–10R-PREBP-short), pHC1-PREBP-full (for p1–10R-PREBP-full), pHC1 (for p1-10R-Mock) and pHC1-No.2 (for p1-10R-No.2) by *Hind*III and inserted into the same restriction site of p1-10R. The direction of the inserted fragments was checked by sequencing analysis.

To obtain the p1-10RΔPRE-PREBP-full plasmid, the same procedure for preparation of p1-10R-PREBP-full was repeated, differing only in that p1cys-Fluc-Δ*cis* [16], which lacks the 102-bp PREBP sequence, was used as the template for the first PCR.

### Stable Transgene Expression in *P. falciparum* and Immuno-Affinity Purification of the Expressed Protein

The plasmids for overexpression were transformed into the parasite by electroporation at 0.310 kV and 975 µF in a 0.2 cm gap cuvette with a Gene Pulser II (Bio Rad, Hercules, CA) as described in a previous study [39]. Drug selection with 25 mg/ml of pyrimethamine was started 48 h after transfection. Parasites that stably expressed the transformed gene, were then selected. For immuno-affinity purification with anti-FLAG antibody, ∼1.5×10^9^ of transgenic parasite-infected erythrocytes were lysed with phosphate-buffered saline (PBS) containing 0.05% saponin. The parasite pellet was washed several times with PBS and diluted with 1000 µl of homogenizing solution (20 mM Tris-HCl (pH 7.5), 150 mM NaCl, 1% NP-40, 100 µg/ml DNaseI, 50 µg/ml RNase, 1 mM PMSF with Protease Inhibitor (Complete Mini, Roche Applied Science, Basel, Switzerland) and lysed by sonication. The lysates were rotated at 15,000 rpm for 30 min, then the supernatants were incubated with 20 µl of ANTI-FLAG M2 Agarose Affinity Gel (Sigma-Aldrich) overnight at 4°C. The protein-bound beads were washed 5 times with wash buffer (50 mM Tris-HCl (pH 8.0), 250 mM NaCl, 10% Glycerol, 0.8% Triton X-100) and then the bound proteins were eluted with the 30 µl of elution buffer (wash buffer with 0.5 mg/ml of the FLAG peptide). The eluents served as immunoprecipitated proteins.

### Immunoprecipitations and Western Blotting

Immunoprecipitations (IP) and Western blotting assays with anti-PREBP peptide antibody were performed with the Rabbit IgG TrueBlot system (eBioscience, SanDiego CA). For the IP experiments, 15 µg of nuclear extract or cytoplasmic fraction from synchronized parasites was diluted with 1 ml of IP buffer (20 mM Tris-HCl (pH 7.5), 150 mM NaCl, 1% NP-40, 1 mM PMSF with Protease Inhibitor (Complete Mini, Roche Applied Science) and precleared by incubation with 50 µl of Protein A Anti-Rabbit IgG beads for 1 h at 4°C. Five µg of anti-PREBP rec. antibody or normal rabbit IgG were coupled to 50 µl of Protein A Anti-Rabbit IgG beads. The beads were incubated with each antibody for 3 h at 4°C and then washed 3 times with 500 µl of wash buffer (50 mM Tris-HCl (pH 8.0), 250 mM NaCl, 10% Glycerol, 0.8% Triton X-100). The precleared nuclear extract was incubated with the antibody-conjugated Protein A Anti-Rabbit IgG beads overnight at 4°C. After washing 5 times with the wash buffer, the bound protein was eluted by boiling with 100 µl of SDS-PAGE sample buffer and 1 µl of eluents were subjected to 5–20% SDS PAGE and Western blotting. Anti-PREBP rec. antibody (2 mg/ml) was used as the primary antibody for Western blotting (1∶5000). HRP-conjugated secondary antibody (secondary antibody Rabbit IgG TrueBlot, eBioscience) was detected using the ECL-Plus reagent (GE Healthcare). For the control Western blotting experiments, each 2 µg of nuclear extract and cytoplasmic fractions were subjected to 5–20% SDS PAGE and blotted onto PVDF membrane. Anti-PfTPx-1 serum was used as the primary antibody for Western blotting (1∶2500) [40]. HRP-conjugated secondary antibody (Cappel) was detected using the ECL-Plus reagent.

### Transient Transfection of *P. falciparum* and Detection of Luciferase Activity

Plasmids were transfected into *P. falciparum* as described previously [16]. In brief, parasites were synchronized with 5% D-sorbitol. Ring-stage parasites (3–10×10^7^) were transfected with 100 µg of plasmid DNA at 0.310 kV and 975 µF in a 0.2 cm gap cuvette with a Gene Pulser II (Bio-Rad). After electroporation, parasites were transferred into a culture dish containing 20 ml RPMI medium and red blood cells at a final hematocrit of 2%. Each transfection cuvette contained 100 µg of pHC1-Rluc, a plasmid expressing Renilla luciferase, as a control for efficiency and recovery. For the luciferase assays, parasite cultures were harvested 20–24 h after transfection at the trophozoite/schizont stage. Each culture was lysed for 5 min at room temperature with 1.0 ml PBS containing 0.15% saponin. Parasite cells were washed twice in PBS, and suspended in 50 µl of passive lysis buffer (Promega, Fitchburg, WI). Firefly and Renilla luciferase activity in parasite extracts were analyzed with a Dual-Luciferase Reporter Assay System (Promega) and Turner luminometer according to the manufacturer’s instructions. The firefly luciferase activity was normalized to that of Renilla luciferase, which was derived from the pHC1-Rluc transfectant.

### Statistical Analysis

Differences were evaluated using Student’s t-test. *P*<0.01 was considered to be statistically significant.

### Accession Numbers

PlasmoDB (www.plasmoDB. org) accession numbers for genes and proteins discussed in this publication are: *pf1-cys-prx*: PF3D7_0802200; PREBP (*P. falciparum*): PF3D7_1011800; PREBP (*P. berghei*): PBANKA_121020; PREBP (*P. yoelii*): PY03523; PREBP (*P. vivax*): PVX_094810; PREBP (*P. knowlesi*): PKH_081130; PREBP (*P. cynomologi*): PCYB_082160. Genbank (www.ncbi.nlm.nih.gov/genbank/) accession numbers for genes and proteins discussed in this publication are: PREBP (*Cryptosporidium parvum*): EAK90228.1; PREBP (*Theileria perve*): EAN34110.1; PREBP (*Bavesia bovis*): EDO07257.1.

## Supporting Information

Figure S1 **Excision of gel slices corresponding to candidate proteins.** Seven µl of final fractions obtained from purification steps were loaded onto the 5–20% SDS-PAGE. The gel was stained with silver-stain kit (SilverQuest, Invitrogen) specialized for mass spectrometry. Three candidate bands (indicated with arrows) were excised as gel pieces and subjected to trypsin digestion and LC-MS/MS analysis. Sizes are indicated in kDa on the right.
(TIF)

Figure S2 **Preparation of the recombinant candidate proteins using the gene cell-free translation system.** A) Immunopurification of the recombinant proteins from cell-free translation reaction mix. The expressed recombinant proteins were eluted from the reaction mixes through immunoprecipitation with the anti-FLAG antibody. The precipitated proteins were eluted with FLAG peptides and 5 µl (∼1 µg) of eluents were subjected to 5–20% SDS-PAGE and blotted onto the PVDF membrane. Blotted proteins were detected with the anti-FLAG antibody. The bands corresponding to the recombinant proteins are indicated with white arrowheads. Protein Nos. 1, 2, 3, 4, 5, 7, 9, 10, 11, 13, 14, 15 and 16 clearly showed the band of the expected molecular weight. Protein No. 12 could not be prepared because the DNA sequence for the gene of protein No. 12 was not amplified by the PCR reaction. Sizes are indicated in kDa at the right of each panel. See Table S2 for detailed descriptions for each protein. B) EMSA with the immunopurified recombinant proteins. The same ^32^P-labeled probe was used in Figure 2 in each assay. Lane N is probe only. EMSA was performed with 2.0 µl (∼0.05 µg) of the fraction of each roughly purified recombinant protein. The reaction mix for EMSA did not contain any nonspecific competitor DNA. Lane P is the positive control of the assay with 0.3 µg of nuclear extract derived from the parasite synchronized at the trophozoite/schizont stage. Positions of the free probe and shifted band corresponding to the PREBP-PRE complex are indicated on the left. No recombinant protein showed any shift-band of comparable size to that of the positive control. Faint shift-bands of the same mobility as those in Lane P, were observed in the lanes 5–16. Such faint bands were sometimes observed with the mock sample (anti-FLAG antibody anticipants from cell-free translation reaction mix without any template mRNA; data not shown), thus, proteins No. 5–16 were judged to not be preferable candidates for PREBP.
(TIF)

Figure S3 **Amino acid sequence of the verified PREBP, PF3D7_1011800.** The deduced molecular weight of PF3D7_1011800 is 132 kDa. The peptides detected in mass spectrometry are indicated with gray shading. The predicted K-homology (KH) domains are indicated with open boxes. The start and end points of the partial ∼60 kDa recombinant protein, which was expressed in the transgenic parasite, are indicated by arrows.
(TIF)

Figure S4 **Patterns of expression of** ***PREBP*** **mRNAs in** ***P. falciparum*** **cells during the erythrocytic stage.** Parasite cultures were tightly synchronized, and parasite-infected erythrocytes were harvested at the indicated times for total RNA extraction and cDNA synthesis. Images of various stages of parasite growth at indicated times are shown in the lower panel. Time 0 corresponds to the final synchronization by D-sorbitol treatment. Real-time quantitative RT-PCR was performed with cDNA templates, and the values recorded were normalized to the amount of 18 S rRNA in each sample measured in the same RT-PCR run. Patterns of expression of *pf1-cys-prx* mRNAs which were measured using the same cDNA sample are indicated as a sub-panel within the main panel.
(TIF)

Figure S5 **Expression level of** ***pf1-cys-prx*** **in parasites that overexpress the central ∼60 kDa region of PREBP.** The parental parasite and the pHC1-No.4 transfectant parasite cultures were synchronized at the trophozoite/schizont stage, and parasite-infected erythrocytes were harvested for total RNA extraction and cDNA synthesis. Real-time quantitative RT-PCR was performed with the cDNA templates and specific primers for *pf1-cys-prx*, and the values recorded were normalized to the amount of 18 S rRNA in each sample measured in the same RT-PCR run. The relative amounts of *PREBP* mRNA were also measured by RT-PCR using same cDNA samples and indicated by the small panel on the right. Data are shown as the means of three independent assays. Error bars represent standard deviations.
(TIF)

Figure S6 **Model scheme for PREBP interaction with PRE sequence and activation of the transcription of** ***pf1-cys-prx*** **.**
(TIF)

Table S1 **Summary of the purification of PREBP from the parasite nuclear extract.**
(DOC)

Table S2 **List of detected** ***P. falciparum*** **proteins by LC-MS/MS.**
(DOC)

Table S3 **List of primers used for the plasmid constructions.**
(DOC)

Method S1 **Preparation of recombinant proteins using the cell free translation system.**
(DOC)

Method S2 **Quantitative real-time PCR.**
(DOC)

## References

[1] World Health Organization (2012) World Malaria Report 2012 (Geneva, Switzerland: World Health Organization).

[2] Richards JS, Beeson JG (2009) The future for blood-stage vaccines against malaria. Immunol Cell Biol 87: 377–390. 19417768 10.1038/icb.2009.27

[3] Aravind L, Iyer LM, Wellems TE, Miller LH (2003) *Plasmodium* biology: genomic gleanings. Cell 115: 771–785. 14697197 10.1016/s0092-8674(03)01023-7

[4] Iyer LM, Anantharaman V, Wolf MY, Aravind L (2008) Comparative genomics of transcription factors and chromatin proteins in parasitic protists and other eukaryotes. Int J Parasitol 38: 1–31. 17949725 10.1016/j.ijpara.2007.07.018

[5] Gutterson N, Reuber TL (2004) Regulation of disease resistance pathways by AP2/ERF transcription factors. Curr Opin Plant Biol 7: 465–471. 15231271 10.1016/j.pbi.2004.04.007

[6] Balaji S, Babu MM, Iyer LM, Aravind L (2005) Discovery of the principal specific transcription factors of Apicomplexa and their implication for the evolution of the AP2-integrase DNA binding domains. Nucleic Acids Res 33: 3994–4006. 16040597 10.1093/nar/gki709 PMC1178005

[7] De Silva EK, Gehrke AR, Olszewski K, León I, Chahal JS, et al (2008) Specific DNA-binding by apicomplexan AP2 transcription factors. Proc Natl Acad Sci U S A 105: 8393–8398. 18541913 10.1073/pnas.0801993105 PMC2423414

[8] Yuda M, Iwanaga S, Shigenobu S, Mair GR, Janse CJ, et al (2009) Identification of a transcription factor in the mosquito-invasive stage of malaria parasites. Mol Microbiol 71: 1402–1414. 19220746 10.1111/j.1365-2958.2009.06609.x

[9] Yuda M, Iwanaga S, Shigenobu S, Kato T, Kaneko I (2010) Transcription factor AP2-Sp and its target genes in malarial sporozoites. Mol Microbiol 75: 854–863. 20025671 10.1111/j.1365-2958.2009.07005.x

[10] Iwanaga S, Kaneko I, Kato T, Yuda M (2012) Identification of an AP2-family protein that is critical for malaria liver stage development. PLoS One 7: e47557. 23144823 10.1371/journal.pone.0047557 PMC3492389

[11] Miller LH, Baruch DI, Marsh K, Doumbo OK (2002) The pathogenic basis of malaria. Nature 415: 673–679. 11832955 10.1038/415673a

[12] Bozdech Z, Llinás M, Pulliam BL, Wong ED, Zhu J, et al (2003) The transcriptome of the intraerythrocytic developmental cycle of *Plasmodium falciparum* . PLoS Biol 1: E5. 12929205 10.1371/journal.pbio.0000005 PMC176545

[13] Le Roch KG, Zhou Y, Blair PL, Grainger M, Moch JK, et al (2003) Discovery of gene function by expression profiling of the malaria parasite life cycle. Science 301: 1503–1508. 12893887 10.1126/science.1087025

[14] Kawazu S, Tsuji N, Hatabu T, Kawai S, Matsumoto Y, et al (2000) Molecular cloning and characterization of a peroxiredoxin from the human malaria parasite *Plasmodium falciparum* . Mol Biochem Parasitol 109: 165–169. 10960175 10.1016/s0166-6851(00)00243-7

[15] Yano K, Komaki-Yasuda K, Kobayashi T, Takemae H, Kita K, et al (2005) Expression of mRNAs and proteins for peroxiredoxins in *Plasmodium falciparum* erythrocytic stage. Parasitol Int 54: 35–41. 15710548 10.1016/j.parint.2004.08.005

[16] Komaki-Yasuda K, Okuwaki M, Kano S, Nagata K, Kawazu S (2008) 5′ sequence-and chromatin modification-dependent gene expression in *Plasmodium falciparum* erythrocytic stage. Mol Biochem Parasitol 162: 40–51. 18692528 10.1016/j.molbiopara.2008.07.002

[17] Grishin NV (2001) KH domain: one motif, two folds. Nucleic Acids Res 29: 638–643. 11160884 10.1093/nar/29.3.638 PMC30387

[18] Valverde R, Edwards L, Regan L (2008) Structure and function of KH domains. FEBS J 275: 2712–2726. 18422648 10.1111/j.1742-4658.2008.06411.x

[19] Epping RJ, Goldstone SD, Ingram LT, Upcroft JA, Ramasamy R, et al (1988) An epitope recognised by inhibitory monoclonal antibodies that react with a 51 kilodalton merozoite surface antigen in *Plasmodium falciparum* . Mol Biochem Parasitol 28: 1–10. 2453800 10.1016/0166-6851(88)90173-9

[20] Hasenkamp S, Russell KT, Horrocks P (2012) Comparison of the absolute and relative efficiencies of electroporation-based transfection protocols for *Plasmodium falciparum* . Malaria J 11: 210. 10.1186/1475-2875-11-210 PMC3407700 22720754

[21] Coulson RM, Hall N, Ouzounis CA (2004) Comparative genomics of transcriptional control in the human malaria parasite *Plasmodium falciparum* . Genome Res 14: 1548–1554. 15256513 10.1101/gr.2218604 PMC509263

[22] Florens L, Washburn MP, Raine JD, Anthony RM, Grainger M, et al (2002) A proteomic view of the *Plasmodium falciparum* life cycle. Nature 419: 520–526. 12368866 10.1038/nature01107

[23] Baldauf SL (2003) The deep roots of eukaryotes. Science 300: 1703–1706. 12805537 10.1126/science.1085544

[24] Siomi H, Matunis MJ, Michael WM, Dreyfuss G (1993) The pre-mRNA binding K protein contains a novel evolutionarily conserved motif. Nucleic Acids Res 21: 1193–1198. 8464704 10.1093/nar/21.5.1193 PMC309281

[25] Duncan R, Bazar L, Michelotti G, Tomonaga T, Krutzsch H, et al (1994) A sequence-specific, single-strand binding protein activates the far upstream element of c-myc and defines a new DNA-binding motif. Genes Dev 8: 465–480. 8125259 10.1101/gad.8.4.465

[26] Braddock DT, Louis JM, Baber JL, Levens D, Clore GM (2002) Structure and dynamics of KH domains from FBP bound to single-stranded DNA. Nature 415: 1051–1056. 11875576 10.1038/4151051a

[27] Kawazu S, Komaki-Yasuda K, Oku H, Kano S (2008) Peroxiredoxins in malaria parasites: parasitologic aspects. Parasitol Int 57: 1–7. 17890140 10.1016/j.parint.2007.08.001

[28] Kimura R, Komaki-Yasuda K, Kawazu S, Kano S (2012) 2-Cys peroxiredoxin of *Plasmodium falciparum* is involved in resistance to heat stress of the parasite. Parasitol Int 62: 137–143. 23201565 10.1016/j.parint.2012.11.005

[29] Fan Q, An L, Cui L (2004) *Plasmodium falciparum* histone acetyltransferase, a yeast GCN5 homologue involved in chromatin remodeling. Eukaryot Cell 3: 264–276. 15075257 10.1128/EC.3.2.264-276.2004 PMC387650

[30] Duffy MF, Selvarajah SA, Josling GA, Petter M (2012) The role of chromatin in *Plasmodium* gene expression. Cell Microbiol 14: 819–828. 22360617 10.1111/j.1462-5822.2012.01777.x

[31] Cui L, Miao J, Furuya T, Li X, Su XZ, et al (2007) PfGCN5-mediated histone H3 acetylation plays a key role in gene expression in *Plasmodium falciparum* . Eukaryot Cell 6: 1219–1227. 17449656 10.1128/EC.00062-07 PMC1951105

[32] Chowdhury I, Mo Y, Gao L, Kazi A, Fisher AB, et al (2009) Oxidant stress stimulates expression of the human peroxiredoxin 6 gene by a transcriptional mechanism involving an antioxidant response element. Free Radic Biol Med 46: 146–153. 18973804 10.1016/reeradbiomed.2008.09.027 PMC2646855

[33] Furey TS (2012) ChIP-seq and beyond: new and improved methodologies to detect and characterize protein-DNA interactions. Nat Rev Genet 13: 840–852. 23090257 10.1038/nrg3306 PMC3591838

[34] Coulson RM, Ouzounis CA (2003) The phylogenetic diversity of eukaryotic transcription. Nucleic Acids Res 31: 653–660. 12527774 10.1093/nar/gkg156 PMC140520

[35] Trager W, Jensen JB (1976) Human malaria parasites in continuous culture. Science 193: 673–675. 781840 10.1126/science.781840

[36] Lambros C, Vanderberg JP (1979) Synchronization of *Plasmodium falciparum* erythrocytic stages in culture. J Parasitol 65: 418–420. 383936

[37] Voss TS, Kaestli M, Vogel D, Bopp S, Beck HP (2003) Identification of nuclear proteins that interact differentially with *Plasmodium falciparum* var gene promoters. Mol Microbiol 48: 1593–1607. 12791141 10.1046/j.1365-2958.2003.03528.x

[38] Crabb BS, Triglia T, Waterkeyn JG, Cowman AF (1997) Stable transgene expression in *Plasmodium falciparum* . Mol Biochem Parasitol 90: 131–144. 9497038 10.1016/s0166-6851(97)00143-6

[39] Fidock DA, Wellems TE (1997) Transformation with human dihydrofolate reductase renders malaria parasites insensitive to WR99210 but does not affect the intrinsic activity of proguanil. Proc Natl Acad Sci U S A 94: 10931–10936. 9380737 10.1073/pnas.94.20.10931 PMC23535

[40] Kawazu S, Komaki K, Tsuji N, Kawai S, Ikenoue N, et al (2000) Molecular characterization of a 2-Cys peroxiredoxin from the human malaria parasite *Plasmodium falciparum* . Mol Biochem Parasitol 116: 73–79. 10.1016/s0166-6851(01)00308-5 11463468

