# Supplementary figures and images for: Identification of a Novel and Unique Transcription Factor in the Intraerythrocytic Stage of Plasmodium falciparum
Source: PLoS One. 2013 Sep 5;8(9):e74701. doi: 10.1371/journal.pone.0074701 (PMC3764013; doi:10.1371/journal.pone.0074701)

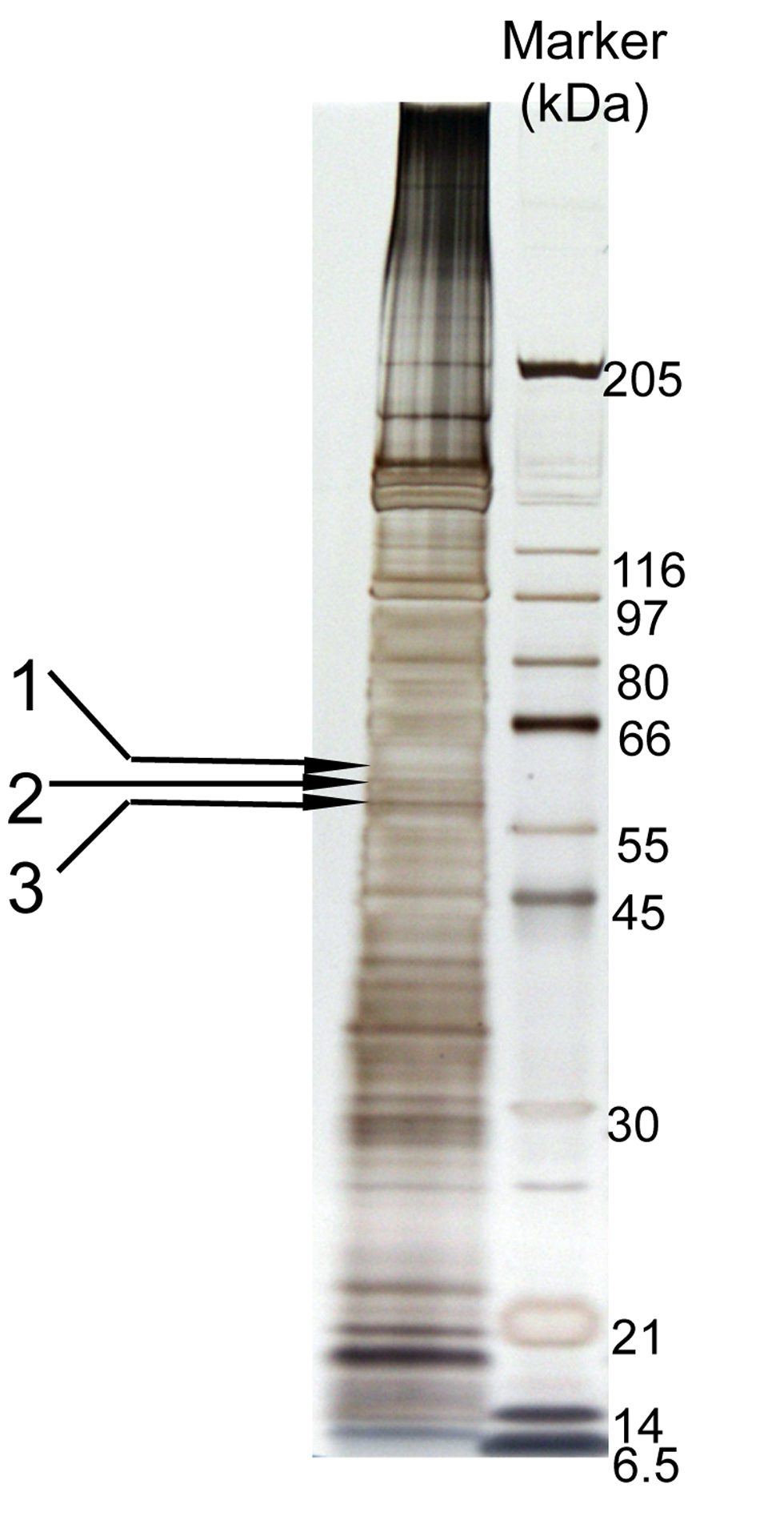

Supplement: Figure S1 — Excision of gel slices corresponding to candidate proteins. Seven µl of final fractions obtained from purification steps were loaded onto the 5–20% SDS-PAGE. The gel was stained with silver-stain kit (SilverQuest, Invitrogen) specialized for mass spectrometry. Three candidate bands (indicated with arrows) were excised as gel pieces and subjected to trypsin digestion and LC-MS/MS analysis. Sizes are indicated in kDa on the right. (TIF) [file pone.0074701.s001.tif]

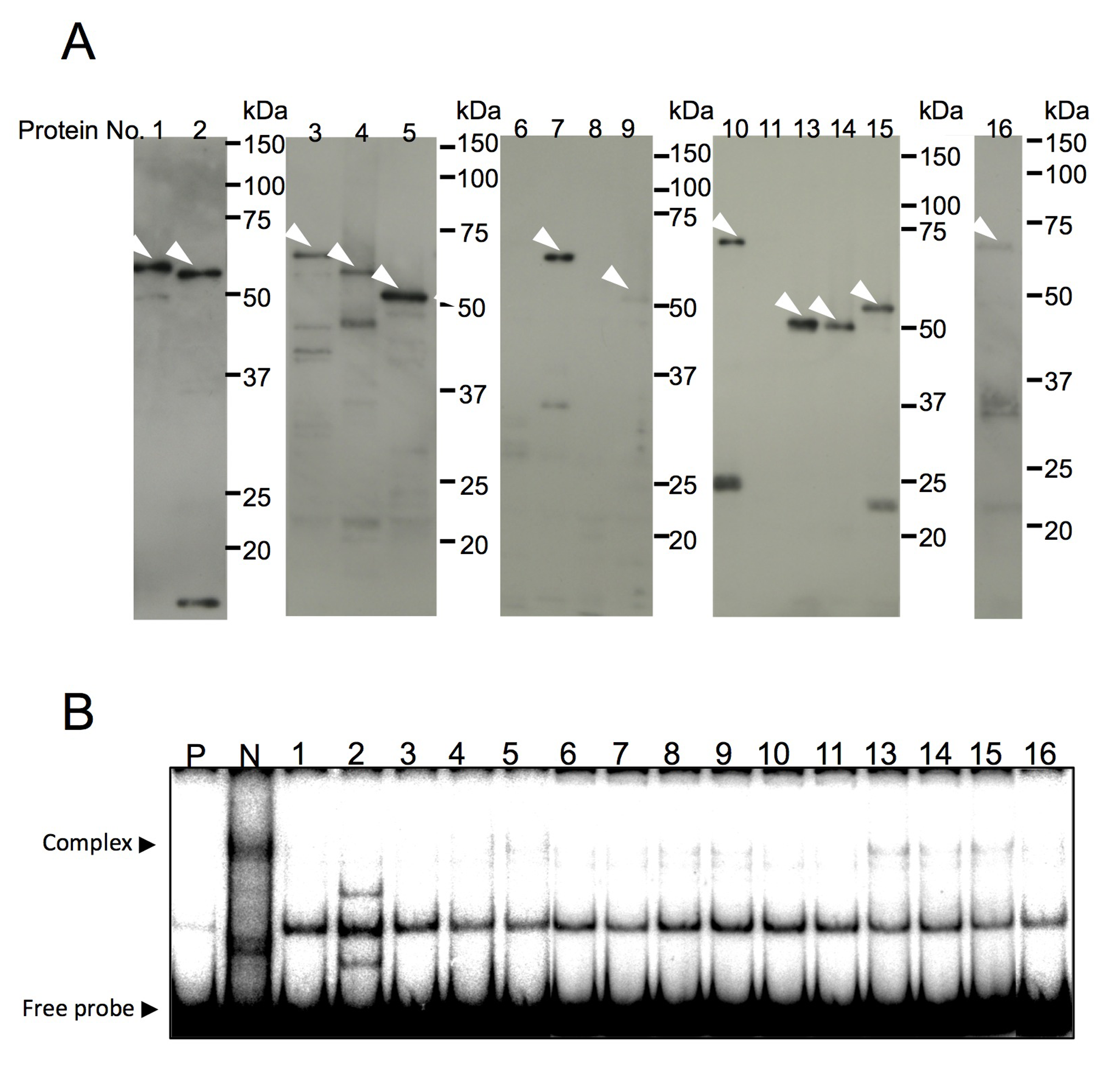

Supplement: Figure S2 — Preparation of the recombinant candidate proteins using the gene cell-free translation system. A) Immunopurification of the recombinant proteins from cell-free translation reaction mix. The expressed recombinant proteins were eluted from the reaction mixes through immunoprecipitation with the anti-FLAG antibody. The precipitated proteins were eluted with FLAG peptides and 5 µl (∼1 µg) of eluents were subjected to 5–20% SDS-PAGE and blotted onto the PVDF membrane. Blotted proteins were detected with the anti-FLAG antibody. The bands corresponding to the recombinant proteins are indicated with white arrowheads. Protein Nos. 1, 2, 3, 4, 5, 7, 9, 10, 11, 13, 14, 15 and 16 clearly showed the band of the expected molecular weight. Protein No. 12 could not be prepared because the DNA sequence for the gene of protein No. 12 was not amplified by the PCR reaction. Sizes are indicated in kDa at the right of each panel. See Table S2 for detailed descriptions for each protein. B) EMSA with the immunopurified recombinant proteins. The same 32P-labeled probe was used in Figure 2 in each assay. Lane N is probe only. EMSA was performed with 2.0 µl (∼0.05 µg) of the fraction of each roughly purified recombinant protein. The reaction mix for EMSA did not contain any nonspecific competitor DNA. Lane P is the positive control of the assay with 0.3 µg of nuclear extract derived from the parasite synchronized at the trophozoite/schizont stage. Positions of the free probe and shifted band corresponding to the PREBP-PRE complex are indicated on the left. No recombinant protein showed any shift-band of comparable size to that of the positive control. Faint shift-bands of the same mobility as those in Lane P, were observed in the lanes 5–16. Such faint bands were sometimes observed with the mock sample (anti-FLAG antibody anticipants from cell-free translation reaction mix without any template mRNA; data not shown), thus, proteins No. 5–16 were judged to not be preferable candidates for PREBP [file pone.0074701.s002.tif]

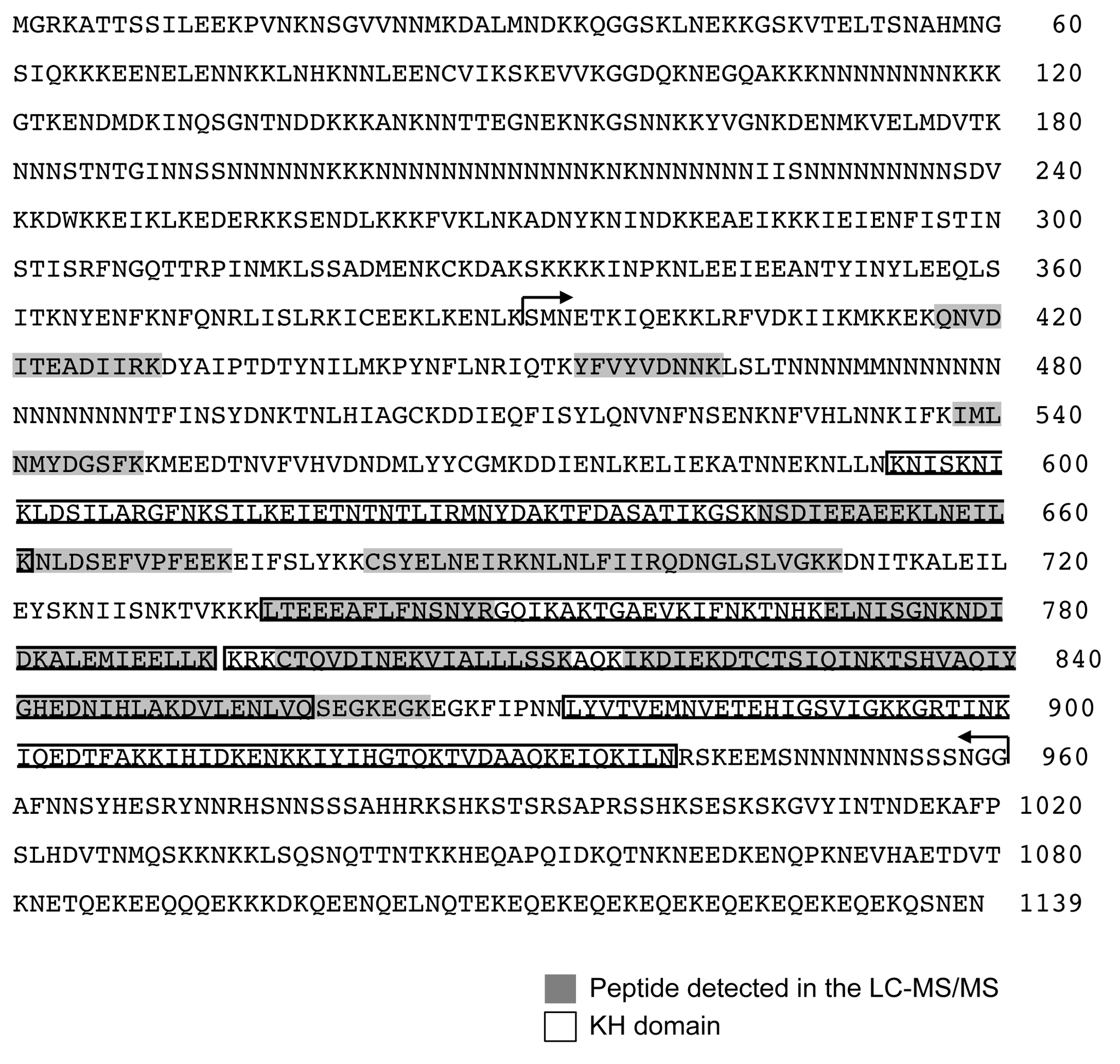

Supplement: Figure S3 — Amino acid sequence of the verified PREBP, PF3D7_1011800. The deduced molecular weight of PF3D7_1011800 is 132 kDa. The peptides detected in mass spectrometry are indicated with gray shading. The predicted K-homology (KH) domains are indicated with open boxes. The start and end points of the partial ∼60 kDa recombinant protein, which was expressed in the transgenic parasite, are indicated by arrows. (TIF) [file pone.0074701.s003.tif]

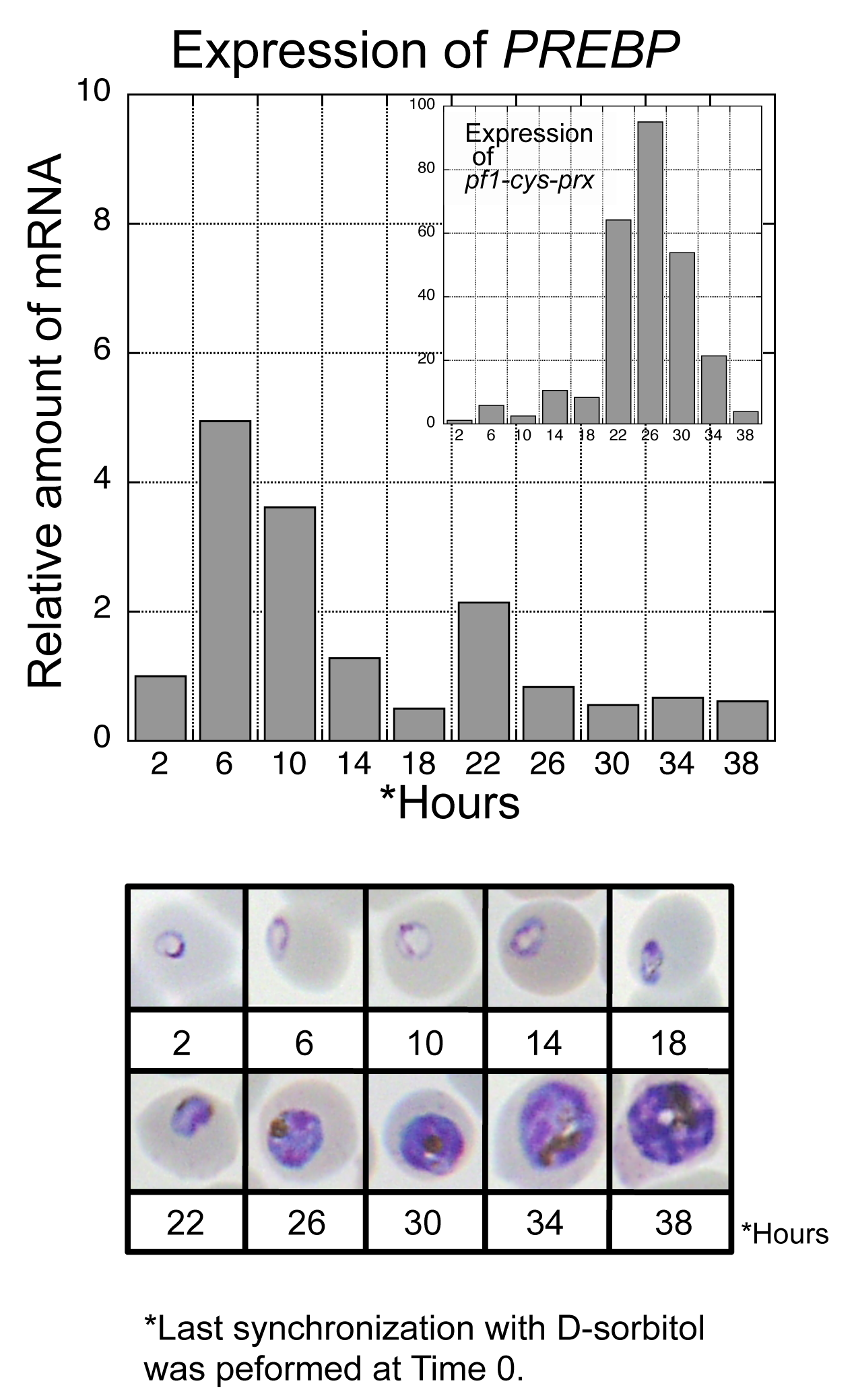

Supplement: Figure S4 — Patterns of expression of PREBP mRNAs in P. falciparum cells during the erythrocytic stage. Parasite cultures were tightly synchronized, and parasite-infected erythrocytes were harvested at the indicated times for total RNA extraction and cDNA synthesis. Images of various stages of parasite growth at indicated times are shown in the lower panel. Time 0 corresponds to the final synchronization by D-sorbitol treatment. Real-time quantitative RT-PCR was performed with cDNA templates, and the values recorded were normalized to the amount of 18 S rRNA in each sample measured in the same RT-PCR run. Patterns of expression of pf1-cys-prx mRNAs which were measured using the same cDNA sample are indicated as a sub-panel within the main panel. (TIF) [file pone.0074701.s004.tif]

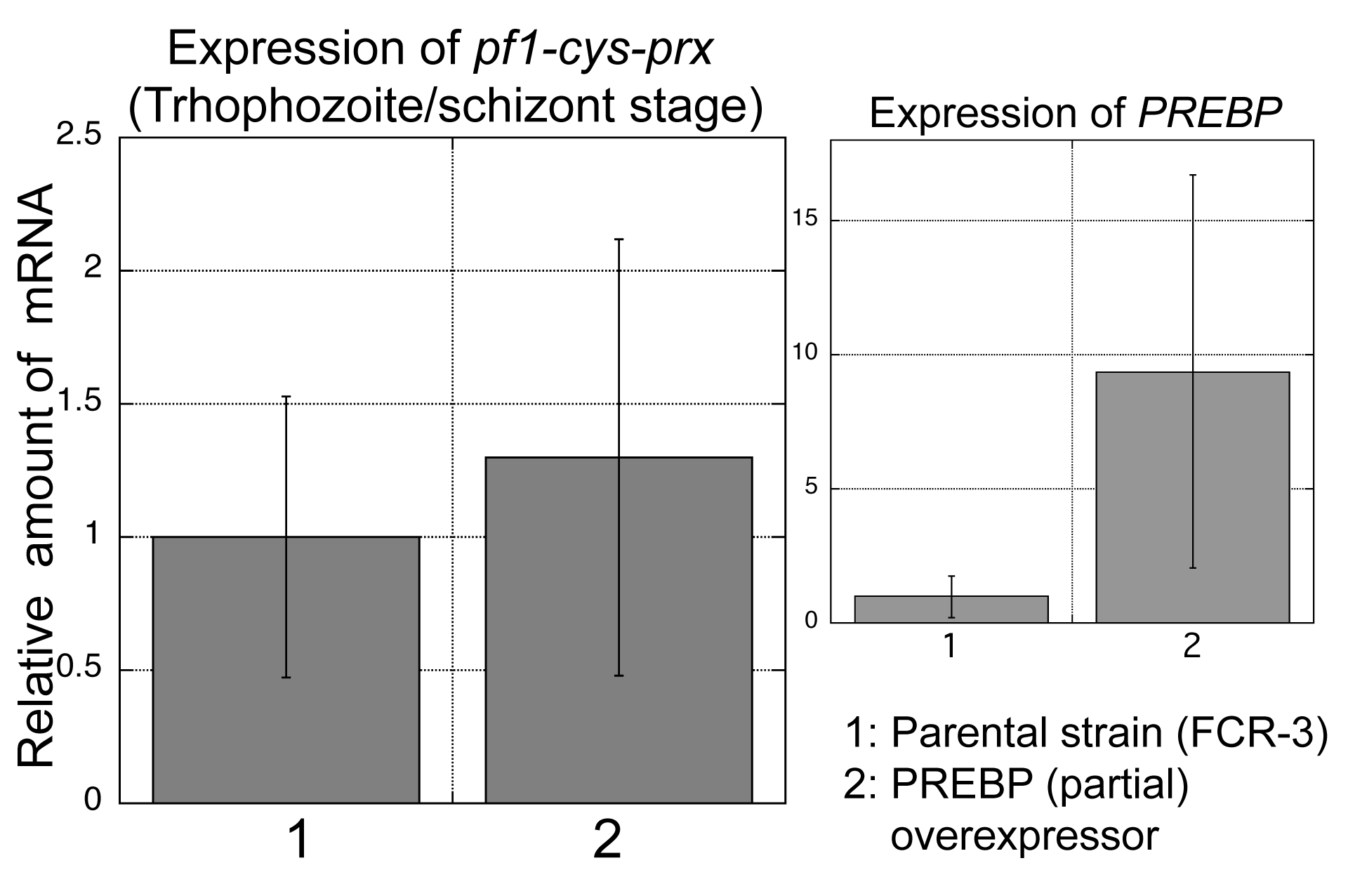

Supplement: Figure S5 — Expression level of pf1-cys-prx in parasites that overexpress the central ∼60 kDa region of PREBP. The parental parasite and the pHC1-No.4 transfectant parasite cultures were synchronized at the trophozoite/schizont stage, and parasite-infected erythrocytes were harvested for total RNA extraction and cDNA synthesis. Real-time quantitative RT-PCR was performed with the cDNA templates and specific primers for pf1-cys-prx, and the values recorded were normalized to the amount of 18 S rRNA in each sample measured in the same RT-PCR run. The relative amounts of PREBP mRNA were also measured by RT-PCR using same cDNA samples and indicated by the small panel on the right. Data are shown as the means of three independent assays. Error bars represent standard deviations. (TIF) [file pone.0074701.s005.tif]

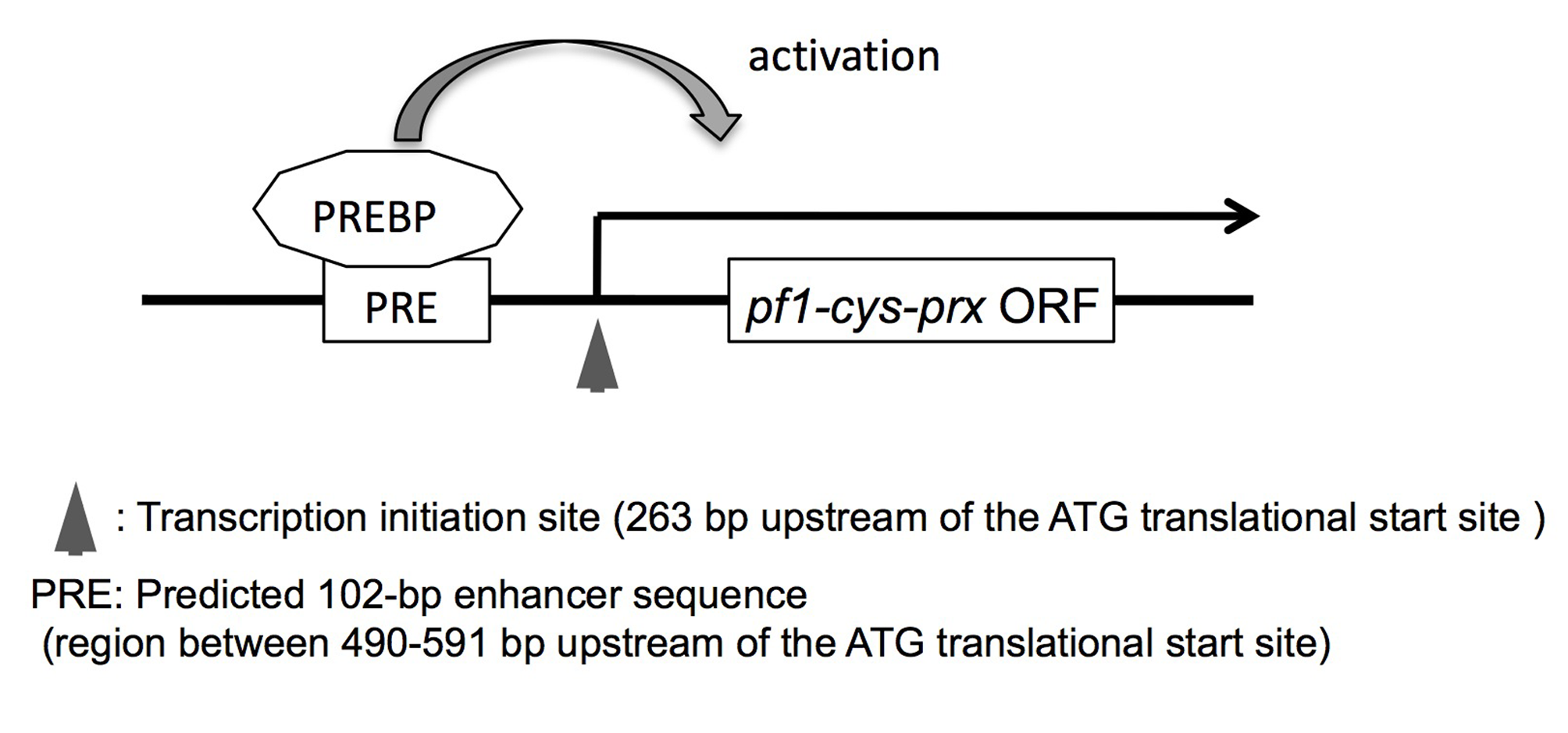

Supplement: Figure S6 — Model scheme for PREBP interaction with PRE sequence and activation of the transcription of pf1-cys-prx . (TIF) [file pone.0074701.s006.tif]
