# Supplementary material for: Identification of a Novel and Unique Transcription Factor in the Intraerythrocytic Stage of Plasmodium falciparum
Source: PLoS One. 2013 Sep 5;8(9):e74701. doi: 10.1371/journal.pone.0074701 (PMC3764013; doi:10.1371/journal.pone.0074701)
Supplement: Table S1 — Summary of the purification of PREBP from the parasite nuclear extract. (DOC) [file pone.0074701.s007.doc]

| Table S1. Summary of the purification of PREBP from the parasite nuclear extract. | | | |
| --- | --- | --- | --- |
|  | Protein conc. (mg/ml) | Volume | Total protein (mg) |
| Nuclear extract^a^ | 0.66 | 110 ml | 73 |
| Step 1: Q Sepharose | 0.36 | 50 ml | 18 |
| Step 2: CM Sepharose | 0.76 | 10 ml | 7.6 |
| Step 3: Resource Phenyl | 0.095 | 11 ml | 1 |
| Step 4: Mono S | ND^b^ | 200 µl | ND |
| Step 5: DNA affinity | ND | 20 µl | ND |
| ^a^ The parasite nuclear extract was prepared from 33 l of culture (corresponding to 5 × 10^11^ parasite cells).  ^b^ ND means “not determined”, due to the scarcity of the purified samples. | | | |
