# Supplementary material for: Identification of a Novel and Unique Transcription Factor in the Intraerythrocytic Stage of Plasmodium falciparum
Source: PLoS One. 2013 Sep 5;8(9):e74701. doi: 10.1371/journal.pone.0074701 (PMC3764013; doi:10.1371/journal.pone.0074701)
Supplement: Table S2 — List of detected P. falciparum proteins by LC-MS/MS. (DOC) [file pone.0074701.s008.doc]

Table S2. List of detected *P. falciparum* proteins by LC-MS/MS.

| **No.** | **MW**^a^ (kDa) | **ID (PlasmoDB)** | **Number of total spectra**^b^ | **Coverage (%)^c^** | **Putative function**^d^ | **Full length (AA)** | **Recombinant Protein**^e^ |
| --- | --- | --- | --- | --- | --- | --- | --- |
| 1 | 61 | PF3D7_0217500 | Band 1: 7, Band 2: 24, Band 3: 3 | 29 | protein kinase | 524 | Full length |
| 2 | 53 | PF3D7_0617200 | Band 1: 2, Band 2: 9, Band 3: 36 | 38 | hypothetical protein (Bfr1p homologue) | 446 | Full length |
| 3 | 129 | PF3D7_1136300 | Band 1: 46, Band 2: 2 | 23 | tudor staphylococcal nuclease (TSN) | 1099 | 281-1098 (518 AA) |
| 4 | 132 | PF3D7_1011800 | Band 1: 16, Band 2: 28, Band 3: 24 | 18 | QF122 antigen | 1139 | 392-960 (569 AA) |
| 5 | 81 | PF3D7_0810600 | Band 1: 9, Band 2: 41, Band 3: 5 | 40 | RNA helicase | 942 | 298-790 (493 AA) |
| 6 | 111 | PF3D7_1216900 | Band 1: 9, Band 2: 5, Band 3: 4 | 11 | DNA-binding chaperone, putative | 940 | 1-600 (600 AA) |
| 7 | 95 | PF3D7_0907600 | Band 1: 11, Band2: 5 | 15 | translation initiation factor SUI1 | 819 | 261-818 (558 AA) |
| 8 | 129 | PF3D7_0513600 | Band 1: 2, Band 2: 5, Band 3: 15 | 13 | deoxyribodipyrimidine photolyase | 1113 | 588-1113 (526 AA) |
| 9 | 84 | PF3D7_1459000 | Band 1: 6, Band 2: 7, Band 3: 15 | 18 | ATP-dependent RNA helicase DBP5 | 741 | 248-741 (494AA) |
| 10 | 128 | PF3D7_1319400 | Band 1: 7, Band 2: 10, Band 3: 17 | 12 | hypothetical protein | 1070 | 11-559 (549 AA) |
| 11 | 73 | PF3D7_1338700 | Band 2: 5 | 9 | hypothetical protein | 622 | Full length |
| 12 | 104 | PF3D7_0207500 | Band 1: 16, Band 2: 13, Band 3: 7 | 21 | serine repeat antigen 6 (SERA6) | 893 | ND (not amplified by PCR） |
| 13 | 49 | PF3D7_1357000 | Band 1: 10, Band 2: 9, Band 3: 11 | 25 | elongation factor 1 alpha | 443 | Full length |
| 14 | 48 | PF3D7_0212300 | Band 1: 4, Band 2: 3, Band 3: 8 | 20 | peptide chain release factor subunit 1 | 427 | Full length |
| 15 | 56 | PF3D7_0406100 | Band 2: 4, Band 3: 6 | 14 | vacuolar ATP synthase subunit b | 494 | Full length |
| 16 | 301 | PF3D7_0903400 | Band 1: 3, Band 2: 6, Band 3: 21 | 7 | DEAD/DEAH box helicase | 2536 | 1961-2536　（576 AA） |

^a^ Deduced molecular weights were calculated from the predicted amino acid sequence of each protein registered in the PlasmoDB.

^b^ Number of total spectra indicates the number of detected peptides from each gel slice. The number of band (from 1 to 3) is according to decreasing molecular weight.

^c^ Coverage (%) indicates he percentage of protein amino acid sequence that is covered by the peptides assigned to the respective database entry.

^d^ Putative function of each protein is indicated according to the prediction of PlasmoDB.

^e^ To investigate the function of each protein the recombinant proteins were prepared using both the parasite cell (Figure 2) and the cell free translation system (Figure S2). In this table, the start and end points of each recombinant protein are indicated as the numbers of amino acids.
