# Supplementary material for: Identification of a Novel and Unique Transcription Factor in the Intraerythrocytic Stage of Plasmodium falciparum
Source: PLoS One. 2013 Sep 5;8(9):e74701. doi: 10.1371/journal.pone.0074701 (PMC3764013; doi:10.1371/journal.pone.0074701)
Supplement: Table S3 — List of primers used for the plasmid constructions. (DOC) [file pone.0074701.s009.doc]

Table S3. List of primers used for the plasmid constructions.

| Name | Sequence (5’-3’) |
| --- | --- |
| FLAG-1F | ATGGACTACAAAGACGATGACGACAAGGGGTGTTCACAAAGTTCAAACGT |
| His-1R | CTAATGGTGATGGTGATGGTGTGAAGATTTATTATCACAAATTTTGTGCA |
| FLAG-2F | ATGGACTACAAAGACGATGACGACAAGACTGAAGCTGAAAATATAAAAATCGA |
| His-2R | CTAATGGTGATGGTGATGGTGAGCCTTAACTTTATTTGCCT |
| FLAG-F | ATGGACTACAAAGACGATGACGACAAGACACCATTAGGTAATAATGA |
| His-3R | CTAATGGTGATGGTGATGGTGATTTTCATCATCATAGTCGATATCT |
| FLAG-s4F | ATGGACTACAAAGACGATGACGACAAGAGTATGAATGAAACTAAAATTCAAGA |
| His-4R | CTAATGGTGATGGTGATGGTGACCACCATTACTACTACT |
| FLAG-5F | ATGGACTACAAAGACGATGACGACAAGATGGAAAAACCTAGATATAAACCT |
| His-5R | CTAATGGTGATGGTGATGGTGCCATGCTTCATTATTATTATCATCT |
| FLAG-6F | ATGGACTACAAAGACGATGACGACAAGAGTGGATGTAATTTAATATGGT |
| His-6R | CTAATGGTGATGGTGATGGTGGTGCACATTTTTAAGATGTCCT |
| FLAG-7F | ATGGACTACAAAGACGATGACGACAAGAAGTCCGAAAAAAAAAGTAATGATAAGA |
| His-7R | CTAATGGTGATGGTGATGGTGACCTTTCGAATTTAAGACT |
| FLAG-8F | ATGGACTACAAAGACGATGACGACAAGAACATAACAAGTAGTAGTCCT |
| His-8R | CTAATGGTGATGGTGATGGTGAGAAGCAACACATTTCTTTTCCCT |
| FLAG-9F | ATGGACTACAAAGACGATGACGACAAGAAGCACAGTGTACTTGTAGAAGA |
| His-9R | CTAATGGTGATGGTGATGGTGATTTTTCAATTTGGTCAAGGATGT |
| FLAG-10F | ATGGACTACAAAGACGATGACGACAAGGAGAACATCACAAGAAAAAGAAACAACCT |
| His-10R | CTAATGGTGATGGTGATGGTGGTTACTATCACATTTGATAAGTGTGT |
| FLAG-11F | ATGGACTACAAAGACGATGACGACAAGGAAAAGTTAAAGGGAGCTCA |
| His-11R | CTAATGGTGATGGTGATGGTG-AAAGTGACTTCTATCCAACTTGACCA |
| FLAG-12F | ATGGACTACAAAGACGATGACGACAAGTCTACAGAACAGCATAGTAGTGT |
| His-12R | CTAATGGTGATGGTGATGGTGTACATAACAGAAATTACAATCTTCTCCCT |
| FLAG-13F | ATGGACTACAAAGACGATGACGACAAGGGTAAGGAAAAAACACATATTAACTTAGT |
| His-13R | CTAATGGTGATGGTGATGGTGTTTTTTGGCTGGTGCTTTAGCTGT |
| FLAG-14F | ATGGACTACAAAGACGATGACGACAAGGAAGACCACGATGCTAATGT |
| His-14R | CTAATGGTGATGGTGATGGTGAAATAATTCGACGTCACTTTCAACATCCT |
| FLAG-15F | ATGGACTACAAAGACGATGACGACAAGAGTAAAGAAGTAGTAAATACAAAAGCTGA |
| His-15R | CTA-ATGGTGATGGTGATGGTGGTTGGCATGGTGACGTGGA |
| FLAG-16F | ATGGACTACAAAGACGATGACGACAAG-GAAGAGGGTAGTTTAAAAAATAAAGT |
| His-16R | CTAATGGTGATGGTGATGGTGATTATTTTTCACATGTAACGAATTGATTGA |
| FLAG-XhoI | CCG CTC GAG ATG GAC TAC AAA GAC GAT GAC GAC AAG |
| His-XhoI | CCG CTC GAG CTA ATG GTG ATG GTG ATG GTG |
| PREBP-F | GCGCTCGAGATGGACTACAAAGACGATGACGACAAGATGGGAAGGAAAGCT ACCA |
| PREBP-R | GCGCTCGAGTTAGAGGCTAGCGTAATCCGGAACATCGTATGGGTAATTTTCATTTGATTGTT |
| ΔHindIII-F | CAAATGATGAAAAGGCTTTCCCAAGTCTTCATGATGTCACCA |
| ΔHindIII-R | TGGTGACATCATGAAGACTTGGGAAAGCCTTTTCATCATTTG |
| FLAG-KpnI | CCGCTCGAGATGGACTACAAAGACGATGACGACAAG |
| His-BamHI | CCGCTCGAGCTAATGGTGATGGTGATGGTG |
| 1-cys5’-F | CGCGGATCCCCTGCAGGTCCAATTGTTGAAATAAGGGTATTCA |
| 1-cys3’-R | CGGGGTACCTGTAAAGGATAGAAGACTATGATTG |
| pTD1 161-179 | GCAGATTGTACTGAGAGTG |
| M13 Reverse2 | GCGGATAACAATTTCACAC |
