# Supplementary material for: Identification of a Novel and Unique Transcription Factor in the Intraerythrocytic Stage of Plasmodium falciparum
Source: PLoS One. 2013 Sep 5;8(9):e74701. doi: 10.1371/journal.pone.0074701 (PMC3764013; doi:10.1371/journal.pone.0074701)
Supplement: Method S1 — Preparation of recombinant proteins using the cell free translation system. (DOC) [file pone.0074701.s010.doc]

**Method S1. Preparation of recombinant proteins using the cell free translation system**

The recombinant proteins for 16 candidate proteins were synthesized with the cell free translation system (TransDirect, Shimazu, Kyoto, Japan). At first, the DNA fragments for the ORF of each gene were amplified by PCR. The primers used for the PCR are indicated in Table S3. Primers FLAG-1F and His-1R were used for the amplification of protein No. 1 and FLAG-2F and His-2R were used for amplification of protein No. 2. The same applied to other candidate proteins. For later purifications, the amplified fragment contained the sequences for FLAG-tag at the 5’ end and for His-tag at the 3’ end. For insertion of these fragments into the pTD1 vector (TransDirect, Shimazu) the *Kpn*I and *Bam*HI sites were added at both 5’ and 3’ end by re-amplification of these fragments by PCR using the primers FLAG-KpnI and His-BamHI. The fragments were inserted into the same restriction site of pTD1. For the preparation of mRNA, template DNA fragments for reverse transcription were amplified with the primers pTD1 161-179 and M13 Reverse 2. 2.5 µg of these DNA fragments were used for the template of the reverse transcription with RiboMAX Express system (Promega). 0.8 µl of the mRNA synthesis reaction mix was used for the cell-free translation reaction in 20 µl of reaction mix of the TransDirect system. The translated proteins were subjected to immuno-affinity purification with ANTI-FLAG M2 Agarose Affinity Gel (Sigma) as described in the main body of this paper.
