# Supplementary material for: Identification of a Novel and Unique Transcription Factor in the Intraerythrocytic Stage of Plasmodium falciparum
Source: PLoS One. 2013 Sep 5;8(9):e74701. doi: 10.1371/journal.pone.0074701 (PMC3764013; doi:10.1371/journal.pone.0074701)
Supplement: Method S2 — Quantitative real-time PCR. (DOC) [file pone.0074701.s011.doc]

**Method S2. Quantitative real-time PCR**

To investigate the mRNA expression profile during intraerythrocytic development, cultures of parasite-infected erythrocytes that were tightly synchronized in the early phase of ring stage through repeated D-sorbitol treatment, and were harvested every 4 h from 2 - 38 h after the final synchronization. This time frame corresponds to the parasite’s development from the early ring stage to the mature schizont. Total RNA was extracted from erythrocyte samples with TRIzol reagent (Life Technologies). Total RNA was reverse transcribed with Ready-To-Go You-Prime First-Strand Beads and pd (N)_6_ random hexamers (GE healthcare).

To compare the gene expression in the transgenic parasites to that in the parental line, cultures of parasite-infected erythrocytes that were synchronized in the trophozoite-shizont stages. Total RNA was extracted from erythrocyte samples with TRIzol reagent. Total RNA was reverse transcribed with Superscript III reverse transcriptase (Life Technologies) and pd (N)_6_ random hexamers.

PCR was performed with the resulting cDNA as a template and sequence-specific oligonucleotide primers for *PREBP*, 5'-CTC GAT TCC ATA TTA GCT AGA GGA-3'　and 5’- CAT TCT TAT CAA TGT GTT TGT GTT GGT TTC TA-3’; for *pf1-cys-prx*, 5’- ATG GCT TACC ATT TAG GAG CTA C -3’ and 5’- CAG CAA GTT CAG TGG TAC AAA CGG GAG -3’. Reaction mixtures were amplified with an ABI PRISM 7900 Sequence Detection System (Life Technologies). Continuous fluorescence observation of amplifying DNA was done with KAPA SYBR Fast qPCR Kit (Nippon Genetics, Tokyo, Japan). To compare the relative amounts of PCR products, fluorescence intensity was recorded by cycle for each amplification and analyzed with the ABI PRISM Sequence Detection System software (Life Technologies). Amplification of the 18S rRNA sequence served as an internal control. The primers used for the amplification of 18S rRNA were 5’-CAA GGA AGT TTA AGG CAA CAA CAG G-3’ and 5’-CAT ATC TTT CAA TCG GTA GGA GCG-3’.
